# From disturbance to resilience: early microbial community establishment in landslide soils of New Zealand

**DOI:** 10.1093/femsec/fiag086

**Published:** 2026-08-03

**Authors:** Olivia Rasigraf, Aaron Bufe, Anja M Schleicher, Dirk Sachse, Niels Hovius, Dirk Wagner

**Affiliations:** GFZ Helmholtz Centre for Geosciences, Section Geomicrobiology, 14473 Potsdam, Germany; Department of Earth and Environmental Sciences, Ludwig Maximilian University of Munich, 80333 Munich, Germany; GFZ Helmholtz Centre for Geosciences, Section Inorganic and Isotope Geochemistry, 14473 Potsdam, Germany; GFZ Helmholtz Centre for Geosciences, Section Geomorphology, 14473 Potsdam, Germany; GFZ Helmholtz Centre for Geosciences, Section Geomorphology, 14473 Potsdam, Germany; Institute of Geosciences, University of Potsdam, 14476 Potsdam, Germany; GFZ Helmholtz Centre for Geosciences, Section Geomicrobiology, 14473 Potsdam, Germany; Institute of Geosciences, University of Potsdam, 14476 Potsdam, Germany

**Keywords:** landslide chronosequence, soil microbial succession, prokaryotic communities, methanotrophs, FAPROTAX, 16S rRNA gene sequencing

## Abstract

Landslides strongly reshape mountain landscapes and initiate soil formation, yet microbial succession in newly formed soils remains poorly understood. We analysed bacterial and archaeal communities along a centennial landslide chronosequence in the Western Southern Alps of New Zealand using 16S rRNA gene amplicon sequencing and geochemical data. A core microbial community was established within ∼3 years and persisted for up to ∼100 years. Alpha diversity declined with soil age and was lowest in the oldest soils (Shannon: 4.8 ± 0.4 to 4.0 ± 0.2). Community composition showed strong and consistent turnover across the chronosequence, with age and soil geochemistry jointly explaining 19% of variation. Bacterial assemblages were dominated by Actinobacteriota, Chloroflexi, and Pseudomonadota, while archaea comprised 3.3%–9.5% of total abundance and were mainly dominated by Thermoproteota and Thermoplasmatota. Increasing soil age was associated with higher total organic carbon, greater abundance of oligotrophic taxa, and higher bacterial 16S rRNA gene copy numbers. Functional predictions indicated a transition from labile carbon turnover in young soils to enhanced potential for complex organic matter degradation in older soils. Overall, microbial communities assemble rapidly after disturbance and undergo gradual, predictable shifts toward more specialized and functionally differentiated assemblages during soil development.

## Teaser

Microbial communities form rapidly in landslide soils and then gradually shift from diverse pioneers to increasingly specialized assemblages as soils develop.

## Introduction

Providing a dynamic interface between the biosphere, atmosphere and the lithosphere, soils represent one of the most critical components of terrestrial ecosystems worldwide (Lal et al. 2018). Globally, soils store over 1500 Gt of organic matter (SOM), representing the largest terrestrial pool of recent organic carbon (OC) and a key component of biogeochemical cycles (Hashimoto et al. 2015, Lal et al. 2018, Crowther et al. 2019).

Soil formation is a complex process driven by interacting biotic and abiotic factors that change throughout ecosystem development. Abiotic factors typically comprise weathering processes when mineral parent material, such as rock or sediment becomes exposed to air and moisture. Physico-chemical processes involving fracturing, leaching and dissolution result in the release of inorganic nutrients and further fragmentation, providing the basis for colonization and activity of micro- and macrobiota (van Breemen 1993, Wojcik et al. 2018). The activity of biota then further accelerates weathering through redox reactions and the release of organic acids, while also contributing to the accumulation of OC and nitrogen (N), and the development of soil structure and slope stabilization through root growth (Wilson and Jones 1983, Hoffland et al. 2004, Rodríguez et al. 2024, Witzgall et al. 2024). Research from different ecosystems has shown that autotrophic and oligotrophic micro-organisms play a critical role at the early stages of soil development when OC and N contents are still low (Crews et al. 2001, Zumsteg et al. 2012, Liu et al. 2016, Khan et al. 2019). These early communities are adapted to nutrient-poor environments and are essential for the initial introduction of OC and reactive forms of N (e.g. N_org_, NH_4_^+^, N oxides) into the soil (Crews et al. 2001).

Our current understanding of soil processes is largely based on research of topo- (catenae) and chronosequences formed on the same parent material in the same ecosystem (Bushnell 1943, Walker et al. 2010). While the main variable in a toposequence is the slope that leads to variations in soil horizons, chronosequences are a series of surfaces on which soils formed under similar boundary conditions but at different starting times (Stevens and Walker 1970, Huggett 1998). Thus, “space for time” substitution in chronosequences allows the study of long-term soil-forming processes (Stevens and Walker 1970, Huggett 1998). Depending on their origin, chronosequences can span from years to millennia (Huggett 1998).

Chronosequences can give insights into the successional dynamics of biota and nutrient accumulation during ecosystem development (Walker et al. 2010, Krauze et al. 2021, Amen et al. 2025). Previous chronosequence research has shown that maturing ecosystems eventually become limited by nutrients, which inevitably leads to regression and the loss of biodiversity (Vitousek et al. 2010). But in contrast to C- and N-scarcity at early stages of development, old soils become limited in phosphorous (P), which is abundant in newly formed substrate (Vitousek et al. 2010). Thus, erosion and exposure of fresh bedrock is an essential process for sustained P supply in ecosystems.

In mountainous terrain, ecosystem renewal and nutrient supply from weathering bedrock is driven by landslides (Walker and Shiels 2012). Depending on local conditions and triggering forces (e.g. precipitation, earthquake ground motion), landslides mobilize regolith and bedrock in varying proportions (Marc et al. 2019). The deposited material can be a mix of bedrock with organic soil and vegetation remnants, which then undergoes a series of physical and chemical alteration processes over the course of succession (Rasigraf and Wagner 2022). The influence of landslides on local geomorphology, weathering, and nutrient dynamics has been extensively studied in different mountainous regions and under various climatic conditions (Schmidt et al. 2008, Hilton et al. 2011, Ramos Scharrón et al. 2012, Emberson et al. 2016, Błońska et al. 2018). Recent landslide deposits are typically limited in OC and reactive N, which, in combination with a loose and unstable surface, is a key limiting factor for colonization by higher plants (Menge and Hedin 2009). Such young landslide deposits, just like other bare soils in initial ecosystems, were shown to become first colonized by pioneer plants which can establish symbiosis with N-fixing bacteria to overcome the N deficiency (Mark et al. 1964, Bergmann et al. 1992). Micro-organisms are typically the first colonizers of barren soils where they contribute to nutrient accumulation through auto- and diazotrophy and accelerate weathering through production of organic metabolites (Berthelin 1988, Amen et al. 2025). The extreme conditions of nutrient limitation and UV exposure also facilitate formation of specialized consortia such as lichens which are widespread in initial ecosystems (Orwin 1970). However, despite the critical role of microbial communities in soil formation, greenhouse gas exchange with the atmosphere, and ecosystem succession (Schlesinger 1997, Bodelier and Steenbergh 2014, Krauze et al. 2021, Rodríguez et al. 2024), their successional dynamics in aging landslide soils remain largely underexplored. While vegetation recovery following landslides has been extensively studied (Walker and del Moral 2003, Walker et al. 2010), much less is known about the trajectories of microbial communities and their functional contribution to ecosystem development. Studies of landslide microbiology can therefore close a knowledge gap on soil development and biogeochemical cycling in mountain ecosystems.

Landslides can be dated with a relatively high precision by using a combination of remote sensing, historic records, and geochronology (Whitehouse and Griffiths 1983, Guzzetti et al. 2012). Therefore, landslides can be used for soil chronosequence studies with a wide age span, depending on their frequency in a given ecosystem and the availability of data. Long-term landslide chronosequences dating back up to several millennia have been used previously for soil research in western China and the Carpathian Mountains (Vindušková et al. 2019, He et al. 2023). At the same time, and in contrast to fluvial, glacial or volcanic chronosequences that typically span centuries to millennia, landslides offer a unique opportunity to investigate soil processes on decadal to centennial timescales with high temporal resolution. This period encompasses the earliest stages of pedogenesis and ecosystem development, when changes in nutrient availability, vegetation establishment, and microbial community assembly are expected to be most rapid (Guariguata 1990, Vindušková et al. 2019).

The Western Southern Alps of New Zealand offer a unique environment where landslide soils can be studied in short-term chronosequences with high resolution. Here, the combination of a super-humid climate with yearly precipitation exceeding 7000 mm, and steep slopes sustained by rapid tectonic uplift sustains conditions for high incidence of landslides (Hovius et al. 1997, Tait et al. 2006). Such extreme climatic conditions not only drive slope instability but also foster rapid weathering and organic matter turnover, making this region an exceptional natural laboratory for studying soil development and microbial influences on its early stages. The average landslide occurrence rate for events of > 10^6^ m^3^ was estimated at 1 per 100 years within the mountain belt, while landslides of smaller volume are far more frequent (Whitehouse and Griffiths 1983, Korup 2005).

In this study, we investigated for the first time the microbial community composition along a landslide deposit chronosequence in the Western Southern Alps of New Zealand, using 16S rRNA gene amplicon sequencing of 180 soil samples collected from 15 landslide deposits and two reference systems. The soils ranged in their deposition age between two and 250 years and were sampled from various depths reaching to 500 cm. By analyzing microbial community succession along this landslide chronosequence, we aimed to (i) characterize changes in prokaryotic community composition across soil development stages, (ii) identify taxa exhibiting broad or restricted ecological distributions, and (iii) evaluate shifts in predicted microbial functional potential associated with ecosystem development.

## Materials and methods

### Study area and sampling

**Figure 1:**
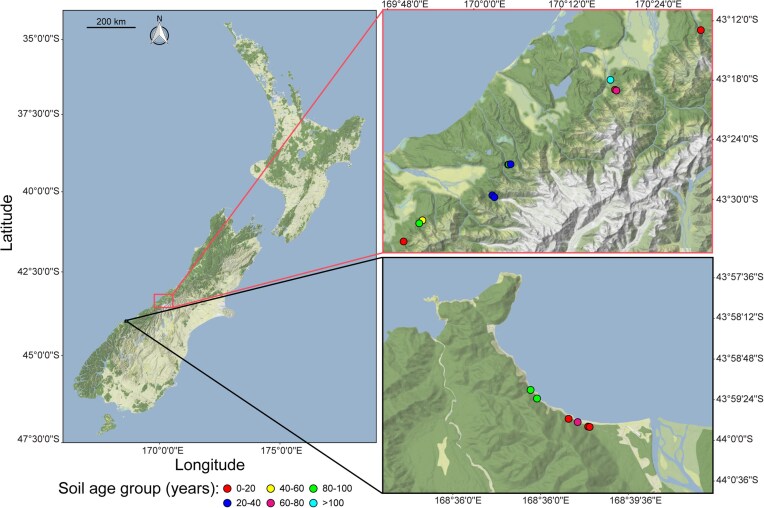
Geographic distribution of sampling sites spanning a centennial landslide chronosequence in the western Southern Alps, New Zealand. Sampling locations represent different stages of post-landslide soil development. Sites on schist bedrock are outlined in red and sites on greywacke bedrock are outlined in black. Base map courtesy of © Stadia Maps, © OpenMapTiles, © Stamen Design, and © OpenStreetMap contributors.

Fifteen landslide deposits ranging in age from 2 to ∼100 years were sampled in November 2017 at six locations along the western flank of New Zealand’s Southern Alps (Fig. 1). The region is characterized by a super-humid climate, with mean monthly precipitation and temperature of 257 ± 32 mm and 11.7 ± 3.3°C during the sampling year, respectively (Franz Josef EWS station, ID 24926). Landslide ages were reconstructed using historical records, satellite imagery, and aerial photographs dating back to 1948, following established approaches for New Zealand landslide chronosequence studies (e.g. Rosser et al. 2017).

Soil material was collected from multiple positions and depths within each deposit to capture horizontal and vertical heterogeneity (Supplementary Table 1). Samples were obtained using ethanol-sterilized spatulas and transferred into sterile 50 ml tubes as field biological replicates (i.e. independent subsamples collected *in situ*). Depending on site morphology, samples were collected either from the deposit surface or from naturally exposed depositional profiles and landslide scarps, providing access to horizons extending to ∼500 cm depth without coring. In total, 180 samples were collected. At two sites (Gaunt Creek: 43°19′02.0“S, 170°19′31.9″E; Hare Mare Creek: 43°26′34.2“S, 170°04′43.2″E), high-resolution depth profiles were sampled at 20 cm intervals down to 80 cm depth.

All samples were transported at 4°C to the GFZ Helmholtz Centre for Geosciences (Potsdam, Germany) and stored at −28°C until molecular and geochemical analyses.

Two reference systems were included: (i) a long-term stable soil at Jackson Bay that had remained unaffected by landsliding for at least 100 years (43°58′47.1″S, 168°36′44.3″E), and (ii) ∼250-year-old fluvial deposits at Gaunt Creek, interpreted as reworked sediments associated with Alpine Fault earthquake-driven mass-movement events (De Pascale and Langridge 2012, Howarth et al. 2018). One Hare Mare Creek deposit (43°26′34.2“S, 170°04′43.2″E), originally dated to 23.5 years, experienced a documented reactivation event during 2013–2014, ∼4.5 years before sampling. Lithology differed among sites, with Jackson Bay soils developed on greywacke bedrock, whereas all other sites originated from Alpine schist-derived material (Supplementary Table 1).

For statistical analyses, samples were assigned to seven age categories (0–20, 20–40, 40–60, 60–80, 80–100, >100-years Gaunt Creek fluvial deposits, and stable soils at Jackson Bay). Sampling intensity varied among categories, with the 0–20-year group contributing the largest number of samples (n = 83), whereas >100-year fluvial deposits and stable soils were represented by fewer samples (n = 8 and n = 6, respectively). Multiple samples were collected within deposits and across locations to account for spatial heterogeneity, and some locations (e.g. Gaunt Creek and Hare Mare Creek) contributed deposits from multiple age categories.

### Geochemical analysis

In the laboratory, water-soluble cations and anions were extracted following the protocol of Blume et al. (2011). Prior to leaching, soil samples were dried at 60°C until constant weight was reached. Subsequently, 5 g of dried soil was mixed with 25 ml of deionized water in 50 ml centrifuge tubes. The suspension was shaken horizontally at 180 r/m for 90 min, centrifuged for 20 min at 9418 g (Sigma 4–16KS, Sigma, Germany), and the supernatant filtered through a 0.22 µm cellulose acetate filter (Minisart, Sartorius Stedim, Germany). Filtered extracts were stored at 4°C until analysis.

Cation concentrations were measured using a Sykam S 155 ion chromatograph with a Sykam S 5200 autosampler (Eresing, Germany). The mobile phase consisted of 0.12 g l⁻¹ methanesulfonic acid and 0.175 g l⁻¹ crown ether at a flow rate of 1.2 ml min⁻¹ using a Reprosol 100 CAT column (Maisch, Germany) at 30°C. Anion concentrations were determined using a Sykam S 155 ion chromatograph with a Sykam S 5300 autosampler (Eresing, Germany), employing 636 mg l⁻¹ Na₂CO₃ and 7.5 mg l⁻¹ NaSCN as eluent at 1 ml min⁻¹ on a SykroGel Ax300 AB-A01 column at 60°C.

Certified multielement standards (ROTIStar, Roth) were used for calibration, and concentrations were calculated from peak areas. All analyses were performed as technical triplicates, and final concentrations were calculated as mean values.

Soil pH was determined for a subset of samples using a 1 : 1 soil-to-water suspension. Approximately 10 g of field-moist soil was mixed with 10 ml of deionized water and shaken at 200 r/m for 1 h at room temperature. After settling, pH was measured in the supernatant using a calibrated pH meter (Mettler Toledo, Switzerland).

#### Elemental composition

Major and minor element concentrations were determined by X-ray fluorescence (XRF) on glass beads prepared from < 62 µm powdered subsamples using an Axios Advanced spectrometer (Malvern PANalytical). Detection limits were 0.02 wt% for major elements and 10 ppm for minor elements. Loss on ignition (LOI) was calculated from mass loss after ignition. Total sulfur (S) was quantified using an Eltra CS 2000 elemental analyzer (Eltra GmbH).

The chemical index of alteration (CIA) was calculated from molar proportions of Al_2_O_3_, CaO (silicate fraction), Na_2_O, and K_2_O following Nesbitt and Young (1982):


\begin{eqnarray*}
{\mathrm{CIA}} = \left[ {{\mathrm{A}}{{\mathrm{l}}}_2{{\mathrm{O}}}_3/\left( {{\mathrm{A}}{{\mathrm{l}}}_2{{\mathrm{O}}}_3 + {\mathrm{CaO}} + {\mathrm{N}}{{\mathrm{a}}}_2{\mathrm{O}} + {{\mathrm{K}}}_2{\mathrm{O}}} \right)} \right] \times 100,
\end{eqnarray*}

to assess the degree of chemical weathering.

#### Total organic carbon (TOC) analysis

Soil subsamples were sieved (<2 mm), dried, and milled at GFZ prior to total organic carbon (TOC) analysis. TOC was determined at the Potsdam Water and Environmental Laboratory (AGROLAB Potsdam GmbH) following DIN EN 15936 (11.12). The analytical precision was < 0.1 g kg⁻¹.

### Microbial community analysis

#### DNA isolation

DNA was extracted in duplicate from soil samples using the FastDNA SPIN Kit for Soil (MP Biomedicals) following the manufacturer’s instructions. Approximately 365–535 mg of soil was used per extraction. DNA was eluted in 100 µl of PCR-grade water. DNA concentrations were quantified fluorometrically using the Qubit dsDNA HS Assay Kit (Invitrogen, Thermo Fisher Scientific). Extracted DNA was stored at −20°C until further analysis.

#### Quantitative polymerase chain reaction (qPCR)

Ribosomal gene copy numbers were quantified by real-time quantitative PCR (qPCR) using SYBR® Green chemistry. Bacterial 16S rRNA genes were amplified with primers eub341F (5′-CCTACGGGAGGCAGCAG-3′) and eub534R (5′-ATTACCGCGGCTGCTGG-3′) (Muyzer et al. 1993). All reactions were performed in technical triplicates using the SensiFAST SYBR NoROX Two-Step Kit (Bioline Reagents, UK) in 96-well optical plates. Amplification was carried out on a CFX Connect Real-Time System (Bio-Rad Laboratories, Germany) using the following cycling conditions: initial denaturation at 95°C for 3 min, followed by 35 cycles of 95°C for 3 s, 60°C for 20 s, 72°C for 30 s, and 80°C for 3 s.

Standard curves were generated from 10-fold serial dilutions of quantified bacterial 16S rRNA gene fragments amplified with primers eub27F (5′-AGAGTTTGATCMTGGCTCAG-3′) (Lane 1991) and eub1492R (5′-GGTTACCTTGTTACGACTT-3′) (Turner et al. 1999). Gene copy numbers were calculated using Bio-Rad CFX Manager 3.1 software (Bio-Rad Laboratories, Germany).

#### 16S RRNA amplification and sequencing

Prokaryotic 16S rRNA genes were amplified using 5′-barcoded universal primers uni515F (5′-GTGYCAGCMGCCGCGGTAA-3′) (Parada et al. 2016) and uni806R (5′-GGACTACNVGGGTWTCTAAT-3′) (Apprill et al. 2015). DNA from each biological sample was pooled from technical duplicates in equimolar concentrations.

PCR reactions (50 µl) contained 5 µl 10× Pol Buffer C (Qiagen, Germany), 2 µl dNTP mix (5 mM each; Qiagen, Germany), 2 µl MgCl₂ (25 mM; Qiagen, Germany), 0.25 µl OptiTaq polymerase (5 U/µl; Qiagen, Germany), 30.75 µl H₂O, 2.5 µl of each primer (100 µM), and 5 µl template DNA. Amplification was performed on a Bio-Rad T100 thermocycler with the following program: initial denaturation at 95°C for 5 min, followed by 35 cycles of 95°C for 30 s, 56°C for 30 s, 72°C for 1 min, and a final extension at 72°C for 7 min.

PCR product quality was verified by gel electrophoresis, purified using AMPure XP beads (Beckman Coulter, USA), and quantified using a Qubit fluorometer (Invitrogen, Thermo Fisher Scientific). Sequencing was performed by GATC Biotech AG using Illumina 2 × 300 bp paired-end sequencing with a 20% PhiX spike-in (v3) to improve sequence diversity. Raw reads were demultiplexed using a custom script based on Cutadapt (Martin 2011).

#### Bioinformatic analysis

Raw paired-end sequences were initially merged using Mothur v1.39.5 (Schloss et al. 2009) with the *make.contigs* function. Resulting contigs were filtered based on overlap length (> 50 bp), maximum mismatches (< 5), and absence of ambiguous bases. Sequence orientation was verified and corrected using QIIME1 (Caporaso et al. 2010), and primer sequences were removed using custom awk scripts.

High-quality sequences were then processed in DADA2 (Callahan et al. 2016), including quality filtering, dereplication, chimera removal, sequence merging, and inference of amplicon sequence variants (ASVs). Taxonomic assignment was performed against the SILVA v138 database (Quast et al. 2012).

#### Taxonomic and statistical analysis

All downstream analyses were conducted in R v4.3.1 (R Core Team 2023) using tidyverse v2.0.0 (Wickham et al. 2019). Raw ASV counts per sample ranged from 7148 to 302 175 reads.

Microbial community data were normalized using ranked subsampling (SRS) implemented in the R package SRS v0.2.3 (Beule and Karlovsky 2020), using the smallest library size (7148 reads) as the C_min_ threshold.

Environmental variables were standardized using z-score transformation prior to multivariate analyses. ASVs were agglomerated at genus level for community-level analyses.

Community composition patterns were explored using Hellinger-transformed genus-level abundances to reduce the influence of zero inflation and compositional effects. For the overall soil community analysis, ordination was performed using non-metric multidimensional scaling (NMDS) based on Bray–Curtis dissimilarities. Group differences were tested using PERMANOVA (vegan v2.6.4; Oksanen et al. 2022) based on Bray–Curtis dissimilarities. Homogeneity of multivariate dispersion was assessed using betadisper to verify that PERMANOVA results were not driven by differences in within-group variance. For analyses of individual landslides and soil depth profiles (Supplementary Fig. 5 and 7), principal coordinate analysis (PCoA) based on Jaccard distances was used to visualize patterns of community turnover. *P*-values were adjusted for multiple testing using the Benjamini–Hochberg procedure where applicable.

Alpha and beta diversity metrics were calculated using phyloseq v1.40.0 (McMurdie and Holmes 2013), microbiome v1.18.0 (Lahti and Shetty 2017), vegan v2.6.4, and ape v5.6.2 (Paradis and Schliep 2019).

To characterize ecological distribution patterns and habitat associations of microbial taxa, two complementary approaches were applied to genus-level data after 0.1% prevalence filtering. Specialist and generalist classification was performed across all samples spanning all age categories. Niche breadth was quantified using Levins’ B index (Levins 1968) implemented in spaa v0.2.2 (Zhang and Zhang 2013), with significance assessed using 999 permutations. Taxa with significantly narrower niche breadth (FDR-adjusted *P* < 0.05) were classified as specialists, while taxa with broader-than-expected niche breadth and *B* ≥ 70% of maximum were classified as generalists. Remaining taxa were considered intermediate. Indicator species analysis was performed on fine-scale depth profiles using indicspecies v1.7.14 (Cáceres and Legendre 2009, De Cáceres and Jansen 2011). All *P*-values were adjusted for multiple testing using the Benjamini–Hochberg procedure. All visualizations were produced using ggplot2 v3.4.2 (Wickham 2016).

#### Functional prediction of microbial communities

Putative prokaryotic functional profiles were inferred from ASV tables using the FAPROTAX database v1.2.10 (Louca et al. 2016) implemented in the R package microeco v1.15.1 (Liu et al. 2021). These assignments represent taxonomically inferred functional potentials rather than directly measured metabolic activities.

Functional annotations were converted into abundance-weighted profiles per sample. ASVs without functional annotation and functions not detected in any sample were removed from further analyses. Functional abundances were log10-transformed [log10(x + 10⁻⁸)] and z-score normalized (row-wise) to ensure comparability across functions and samples.

Ecologically relevant functions were grouped *a priori* into carbon, nitrogen, methane, sulfur cycling, and phototrophy categories. Functions not detected in the dataset were excluded from further analysis.

Functional profiles were aggregated by soil age group category using mean values. Heatmaps were generated using pheatmap v1.0.12 with hierarchical clustering.

#### Sequence data repository

All 16S rRNA amplicon sequencing data generated in this study have been deposited in the European Nucleotide Archive (ENA) under project number PRJEB39192 (ERP122683), with the project title “Primary succession of soil microbial communities in landslide chronosequence in the Southern Alps of New Zealand.”

## Results

### Soil mineralogy, soil pH, and bacterial abundance

**Figure 2:**
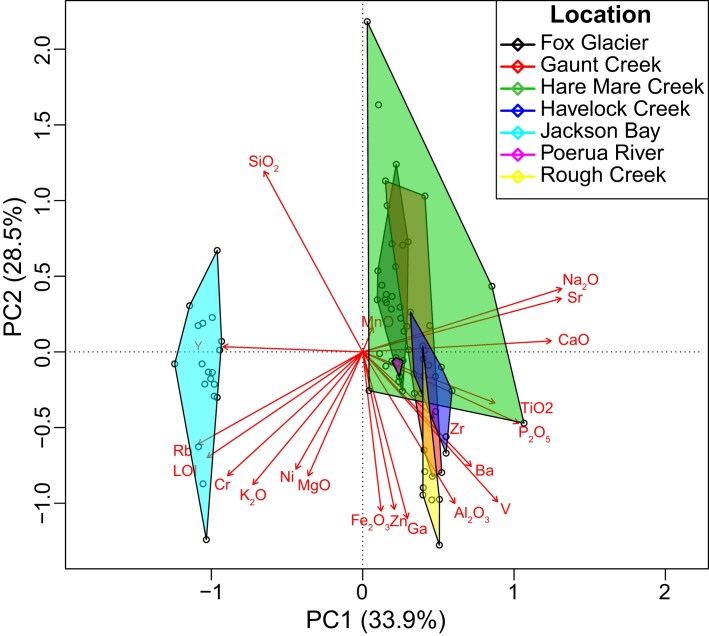
Principal component analysis showing variation in soil elemental composition among landslide locations. Soil elemental composition was determined by X-ray fluorescence (XRF). All landslides are situated in schist of the Torlesse Terrane (predominantly greywacke protolith), except those at Jackson Bay, which occur in unmetamorphosed greywacke bedrock. Abbreviations: LOI, loss on ignition; TOC, total organic carbon.

Elemental analysis revealed a clear separation of soils according to underlying bedrock chemistry (Fig. 2). CIA values ranged from 57.07 ± 1.60 to 63.04 ± 0.36 across the six sites developed on Alpine schist, whereas Jackson Bay soils developed on greywacke exhibited consistently higher CIA values (72.97 ± 1.73). Within each bedrock type, CIA did not vary significantly with landslide age, consistent with previous studies indicating that early weathering is initially dominated by carbonate and sulfide depletion, whereas silicate weathering becomes increasingly important during later stages without substantially altering bulk CIA values (Emberson et al. 2016).

Principal component analysis (PCA) further separated soils according to parent material. Greywacke-derived soils from Jackson Bay were associated with higher concentrations of Rb, Cr, K_2_O, and loss on ignition (LOI), whereas schist-derived soils were characterized by higher Sr, CaO, Na_2_O, and P_2_O_5_.

Soil pH exhibited considerable variation across the chronosequence but was measured only for a subset of samples, resulting in uneven representation among age categories. Young landslide deposits showed a broad pH range, from moderately acidic to alkaline conditions (5.6–8.8), whereas older, organic-rich soils consistently exhibited acidic conditions with substantially lower variability (typically pH 5.1–6.3). Across all measured samples, pH was negatively correlated with TOC (Spearman’s *ρ* = −0.64, *P* < 0.001), indicating increasing acidification with organic matter accumulation during soil development (Supplementary Fig. 1). In contrast, low-TOC soils displayed a much broader pH range, suggesting more variable geochemical conditions during early stages of succession.

**Figure 3:**
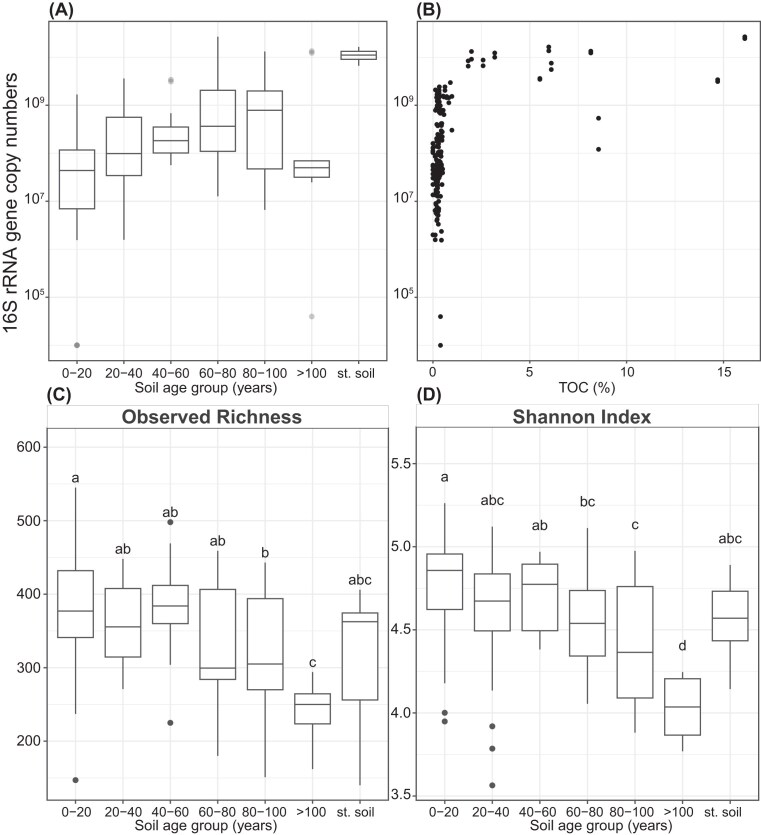
Bacterial abundance, alpha diversity, and total organic carbon across the landslide soil chronosequence. (A) Distribution of bacterial 16S rRNA gene copy numbers across soil age groups. (B) Relationship between bacterial 16S rRNA gene copy numbers and total organic carbon (TOC). (C) Observed richness across soil age groups. (D) Shannon diversity across soil age groups. Different letters indicate significant differences among soil age groups based on pairwise Wilcoxon tests (adjusted *P* < 0.05), whereas identical letters indicate no significant difference. Abbreviation: st. soil, stable soil.

Grouping samples by landslide age further revealed a progressive increase in bacterial 16S rRNA gene copy numbers along the chronosequence. Mean abundances increased by approximately one order of magnitude, from 3.39 × 10⁸ ± 5.87 × 10⁸ copies g⁻¹ soil in the 0–20-year age group to 2.32 × 10⁹ ± 3.43 × 10⁹ copies g⁻¹ soil in the 80–100-year group (Fig. 3a). This pattern was consistent across landslide locations irrespective of parent material. Bacterial 16S rRNA gene abundance was positively correlated with TOC (Spearman’s *ρ* = 0.60, *P* < 0.001), indicating increasing bacterial biomass with progressive soil development (Fig. 3b).

### Landslide soil microbial community structure and diversity

A total of 96 145 ASVs were retained after quality filtering, including 93 619 bacterial ASVs and 2 496 archaeal ASVs. Taxonomic agglomeration at genus level resulted in 1776 genera. Soils from all age groups shared a total of 314 genera, which comprised the core community.

Observed richness and Shannon diversity were highest in the youngest soils (0–20-year) and generally decreased with increasing soil age (Fig. 3c and d). Pairwise Wilcoxon rank tests confirmed that richness in the 0–20-year soils was significantly higher than in the 80–100-year group (*P* < 0.01) and the Gaunt Creek fluvial deposit (*P* < 0.001), while differences compared with intermediate age groups (20–40-, 40–60-, 60–80-year) were not significant. Shannon diversity showed a similar pattern, with significantly higher values in the 0–20-year soils (4.8 ± 0.4) compared to the 60–80-year (4.5 ± 0.3, *P* = 0.019), 80–100-year (4.4 ± 0.4, *P* < 0.001), and Gaunt Creek fluvial deposit (4.0 ± 0.2, *P* < 0.001). Differences with stable soils at Jackson Bay were largely non-significant, except for the Gaunt Creek fluvial deposit, which displayed slightly higher Shannon diversity than the Jackson Bay reference (*P* = 0.014). Together, these results indicate a progressive decline in microbial richness and evenness with increasing soil age along the chronosequence.

For further comparison of microbial community composition across soil age groups, non-metric multidimensional scaling (NMDS) was performed using Bray–Curtis dissimilarities of Hellinger-transformed community data (Supplementary Fig. 2A). Communities from most soil groups showed substantial overlap in ordination space.

Because the >100-year Gaunt Creek fluvial deposit and the mature stable soils at Jackson Bay do not represent successional stages within the landslide chronosequence, age-related community turnover was evaluated separately using only the five chronosequence age classes (0–20-, 20–40-, 40–60-, 60–80- and 80–100-year; Supplementary Fig. 2B). Pairwise PERMANOVA analyses detected significant differences between all age groups (adjusted *P* ≤ 0.02), with effect sizes ranging from *R²* = 0.049 to 0.131. The strongest compositional shifts occurred between the 20–40- and 40–60-year soils (*R²* = 0.131) and between the 20–40- and 60–80-year soils (*R²* = 0.111), whereas the smallest difference was observed between the 60–80- and 80–100-year groups (*R²* = 0.056). These results indicate progressive but gradual turnover of microbial communities during soil development, with evidence for increasing community stabilization in later successional stages.

Tests for multivariate dispersion showed a modest but significant effect of age category (permutational test, *P* = 0.03), indicating differences in within-group variability among age classes. However, the persistence of significant pairwise differences suggests that compositional turnover, rather than dispersion alone, dominates community differentiation across the chronosequence.

A PERMANOVA model including environmental variables further indicated that microbial community composition was jointly influenced by successional age and soil geochemistry (*R²* = 0.353, *P* = 0.001), although age and location effects were partially confounded due to uneven sampling across sites.

To disentangle the relative contributions of environmental drivers to microbial community composition, a distance-based redundancy analysis (dbRDA) was performed using age category and key geochemical variables. The constrained model explained 18.98% of total variation in Bray–Curtis dissimilarities. The overall model was significant (permutation test, *P* = 0.001), as were all included predictor variables, with the exception of CIA and NO₃⁻, which showed only marginal effects. Among individual predictors, age category was a significant structuring factor (*P* = 0.001), together with TOC (*P* = 0.001), SO_4_²⁻ (*P* = 0.001), and Ca²⁺ (*P* = 0.001), indicating that both successional stage and soil chemistry contribute to shaping microbial community composition.

Variation partitioning revealed that age category alone explained 4.2% of the variation (adjusted *R²*), while soil geochemistry accounted for 5.2%, with a small shared fraction of 0.6%. The remaining variation (90.1%) was unexplained, indicating that additional factors such as microscale heterogeneity, unmeasured environmental variables, and stochastic assembly processes likely contribute substantially to community variation.

#### Bacterial community

**Figure 4:**
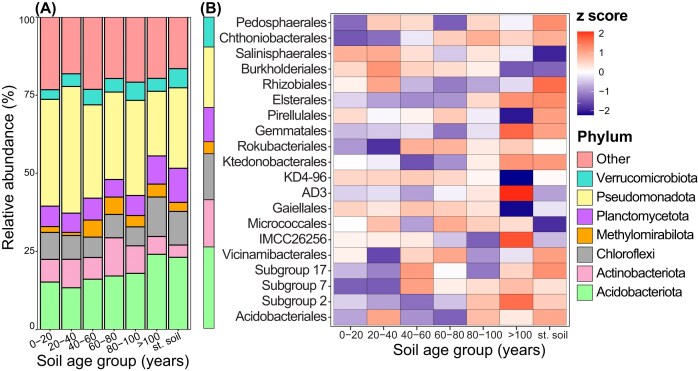
Bacterial community composition across soil age groups in the western Southern Alps of New Zealand. (A) Relative abundances of the seven most abundant bacterial phyla. (B) Heatmap showing row-wise standardized (z-score) relative abundances of the 20 most abundant bacterial orders. Z-scores were calculated separately for each order across all samples, such that values represent the number of standard deviations a sample’s relative abundance deviates from the mean relative abundance of that order. Data represent mean relative abundances of samples within each soil group. Groups comprise 0–20-, 20–40-, 40–60-, 60–80-, and 80–100-year landslide soils, the >100-year Gaunt Creek fluvial deposit, and stable soils from Jackson Creek (“st. soil”).

Across all soils, bacterial communities were consistently dominated by the phyla Acidobacteriota, Actinobacteriota, Chloroflexi, Methylomirabilota, Planctomycetota, Pseudomonadota (formerly Proteobacteria), and Verrucomicrobiota, which together accounted for 77%–84% of the total prokaryotic community (Fig. 4A).

Pseudomonadota were most abundant in early successional soils, reaching 34.6 ± 9.8% (0–20-year group) and 40.5 ± 10.1% (20–40-year group), and declined in older systems, including the Gaunt Creek fluvial deposit (20.7 ± 5.1%) and stable soils at Jackson Bay (25.7 ± 3.7%). This pattern was driven mainly by Burkholderiales and Salinisphaerales, whereas Alphaproteobacteria (notably Rhizobiales and Elsterales) increased in later stages, reaching highest abundances in stable soils and the fluvial deposit (Fig. 4B).

Acidobacteriota increased significantly in older soils (18.0 ± 6.9% in 80–100-year group; 24.1 ± 3.1% in the Gaunt Creek fluvial deposit; *P* < 0.001), mainly due to Acidobacteriota Subgroup 2, while other subgroups showed more variable distributions. Planctomycetota and Verrucomicrobiota also increased with soil age, reaching up to 11% and 6%, respectively, in late successional stages.

Methylomirabilota peaked in intermediate-aged soils (up to 5%), largely associated with Rokubacteriales. Chloroflexi were most abundant in the fluvial deposit (13%) and stable soils at Jackson Bay (11%), while Actinobacteriota remained relatively stable, with only lineage-specific shifts (e.g. decline of Gaiellales in older deposits and slight enrichment of IMCC26256 in the Gaunt Creek fluvial deposit).

Overall, bacterial succession was characterized by a shift from Pseudomonadota-dominated early communities toward increasing contributions of Acidobacteriota, Chloroflexi, Planctomycetota, and Verrucomicrobiota in later successional stages.

#### Archaeal community

**Figure 5:**
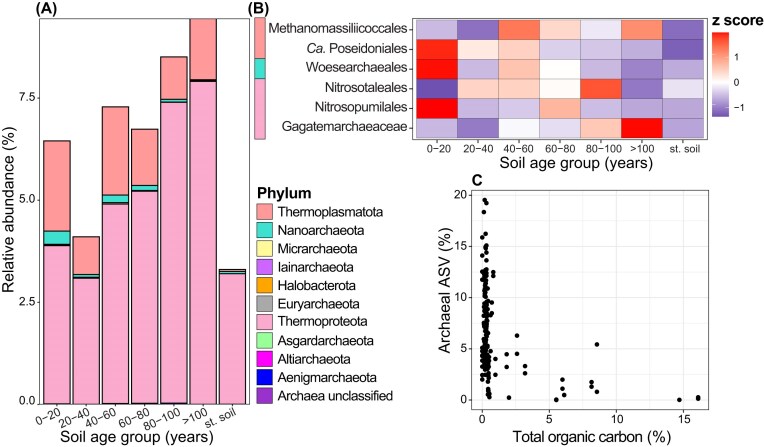
Archaeal community composition across soil age groups in the western Southern Alps of New Zealand. (A) Relative abundances of all detected archaeal phyla. (B) Heatmap showing row-wise standardized (z-score) relative abundances of the six most abundant archaeal orders. Z-scores were calculated separately for each order across all samples, representing the number of standard deviations a sample’s relative abundance deviates from the mean of that order. (C) Relationship between archaeal relative abundance and total organic carbon (TOC). Data represent mean relative abundances within each soil group. Groups comprise 0–20-, 20–40-, 40–60-, 60–80-, and 80–100-year landslide soils, the >100-year Gaunt Creek fluvial deposit, and stable soils from Jackson Creek (“st. soil”).

Archaeal ASVs were detected in all sampled soils, with SRS-normalised relative abundances ranging from 3.3% in stable soils at Jackson Bay to 9.5% in the Gaunt Creek fluvial deposit. Overall, archaeal relative abundance showed a tendency to increase with soil age, but was significantly negatively correlated with soil TOC content (Fig. 5A and C, Supplementary Fig. 3).

Archaeal community composition was broadly similar across locations and age groups (Fig. 5). The dominant archaeal lineages corresponded to the phyla Thermoproteota (formerly Crenarchaeota), Thermoplasmatota, and Nanoarchaeota. Lineages commonly associated with methanogenesis within Euryarchaeota were detected only at very low relative abundances (< 0.1%) in a small subset of samples.

Within Thermoplasmatota, ASVs assigned to Methanomassiliicoccales were present across most soils. Their average relative abundance ranged between 1–2% in most age groups, with substantially lower values (< 0.1%) in stable soils at Jackson Bay. The second most abundant lineage, *Ca*. Poseidoniales (formely Marine Group II) reached up to 1.7% in the youngest (0–20-year group) soils and declined to low or undetectable levels in older stable soils.

Nanoarchaeotal ASVs were represented almost exclusively by Woesearchaeales, which followed a similar distribution pattern to *Ca*. Poseidoniales, with highest relative abundances in the youngest soils and lowest values in the Gaunt Creek fluvial deposit. Overall, Nanoarchaeota remained consistently low across all samples (< 0.3%).

Thermoproteota was the most abundant archaeal phylum overall, reaching up to 7.4% in 80–100-year group and 7.9% in the Gaunt Creek fluvial deposit. Within this group, the orders Nitrosotaleales, Nitrosopumilales, and lineages affiliated with the family Gagatemarchaeaceae (formerly Group 1.1c) were dominant.

Nitrosopumilales were most abundant in the youngest soils (0–20-year group), whereas Nitrosotaleales were consistently detected across all soils (2–4% relative abundance). Gagatemarchaeaceae reached their highest relative abundance (> 5%) in the Gaunt Creek fluvial deposit.

#### Specialist/generalist taxa analysis

To further explore taxon-level ecological specificity across soil age groups, we performed a specialist/generalist analysis based on niche breadth (Levins’ B index). Across all defined soil age groups, 122 taxa were classified as specialists and 66 as intermediates, while no true generalists were detected, indicating that the chronosequence imposes strong selective pressures favoring niche-adapted organisms (Supplementary Fig. 4). Specialists were generally among the most abundant taxa, including Subgroup 2 (ASV_56), and uncultured members of Vicinamibacterales (ASV_13) and Gemmataceae (ASV_109). Their distributions followed clear age-dependent patterns: the youngest soils (0–20-year group) were dominated by ASV_13 (3.57%) and ASV_109 (3.22%), intermediate-aged soils (20–80-year groups) by *Massilia* (ASV_8; 5.18%) and Rokubacteriales (ASV_19; 4.83%), and the Gaunt Creek fluvial deposit by Subgroup 2 (ASV_56; 11.75%), unclassified Gemmataceae (ASV_109; 7.21%), and Gagatemarchaeaceae (ASV_30; 5.70%).

Intermediate taxa represented more environmentally resilient members of the community, with the most abundant being Nitrosotaleaceae (ASV_34; 2.38%), uncultured genera within Acidobacteriales (ASV_165; 2.13%), and Subgroup 7 (ASV_18; 1.71%). Their presence across multiple soil ages suggested a broader ecological tolerance compared to specialists.

### Vertical distribution of microbial communities in landslide soils

Vertical differentiation of microbial communities in landslide-affected soils is expected to depend on the site’s depositional history, stabilization state, and organic matter turnover. Across the studied chronosequence, depth profiles revealed both homogeneous and stratified patterns, suggesting that microbial vertical organization is strongly modulated by local geomorphic and biogeochemical conditions. To illustrate the range of observed variability, two contrasting examples are presented below in higher vertical resolution.

**Figure 6:**
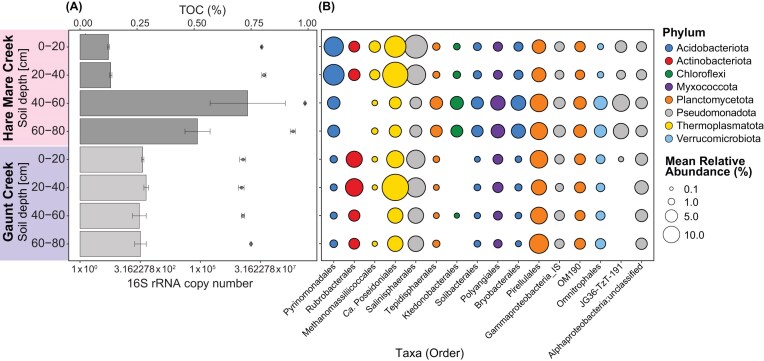
Microbial community composition in two soil depth profiles from landslide deposits in the Southern Alps of New Zealand. Profiles from a 4.5-year-old landslide (Hare Mare Creek) and a 12.5-year-old landslide (Gaunt Creek) are shown. (A) Total organic carbon (horizontal bars) and 16S rRNA gene copy numbers (points) across soil depth. Data points represent means of duplicate samples, with error bars indicating standard errors. (B) Mean relative abundances of prokaryotic orders significantly associated with shallow (0–40 cm) and deep (40–80 cm) soil layers at the Hare Mare Creek site, identified by indicator species analysis (IndVal > 0.9). Only taxa with relative abundances > 0.5% are shown.

At the 4.5-year-old landslide at Hare Mare Creek, a marked separation in microbial community composition was observed at 40 cm depth (Supplementary Fig. 5), closely associated with changes in TOC content and 16S rRNA gene copy numbers (Fig. 6A). Indicator species analysis at a 0.1% prevalence cutoff identified distinct prokaryotic lineages associated with specific depth intervals. To highlight ecologically relevant taxa, we focus on those with > 0.5% relative abundance. At the order level (IndVal > 0.9), the upper profile (0–40 cm) was significantly associated with Pyrinomonadales (Acidobacteriota), Rubrobacterales (Actinobacteriota), Salinisphaerales (Pseudomonadota), as well as archaeal lineages such as Methanomassiliicoccales and *Ca*. Poseidoniales (Fig. 6B). In contrast, the lower profile (40-80 cm) was characterized by indicators from Tepidisphaerales, Pirellulales, OM190, Solibacterales, Bryobacterales, Ktedonobacterales, Polyangiales, Omnitrophales, and additional pseudomonadotal lineages including Gammaproteobacteria incertae sedis, JG36-TzT-191, and unclassified Alphaproteobacteria. These indicator patterns underscore that, despite overall vertical homogeneity, discrete horizons in individual landslides can harbor distinct microbial lineages linked to local depositional and geochemical conditions.

In contrast, the 12.5-year-old landslide at Gaunt Creek showed no such apparent depth-related separation in either microbial composition or geochemical parameters (Supplementary Fig. 5).

### Predicted prokaryotic functional profiles

FAPROTAX assigned putative ecological functions to 35.2% of total sequence reads across all samples. Functional mapping efficiency varied among samples, ranging from 12.4% to 65.6%, but showed broadly comparable values across soil age categories (mean range: 28.6%–39.5%). In total, 73 functional groups were detected. Functional assignments represent taxonomically inferred ecological potentials and do not provide direct evidence of gene presence, gene expression, or metabolic activity.

**Figure 7:**
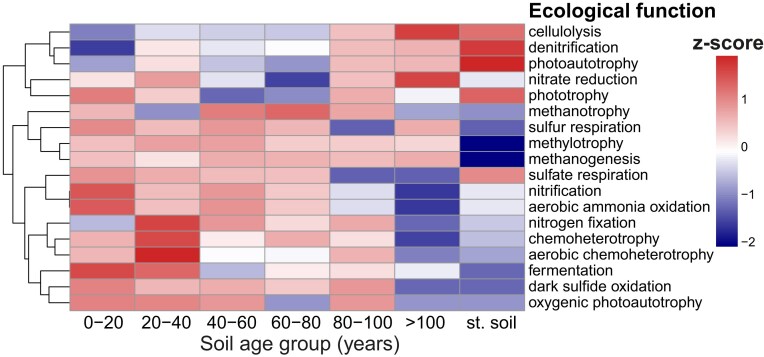
Predicted microbial functional profiles across the landslide soil chronosequence. Heatmap showing row-wise standardized (z-scores) of mean relative abundances of microbial functions predicted using FAPROTAX from 16S rRNA gene amplicon data. Values represent relative enrichment or depletion of predicted functional abundances compared with the mean abundance of each function across all soil groups. Functions are grouped into major biogeochemical categories. Hierarchical clustering was applied to functions (rows), while soil groups (columns) are ordered chronologically. Groups comprise 0–20-, 20–40-, 40–60-, 60–80-, and 80–100-year landslide soils, the >100-year Gaunt Creek fluvial deposit, and stable soils from Jackson Creek (“st. soil”).

FAPROTAX-based predictions indicated shifts in the relative representation of major biogeochemical functions across the landslide chronosequence (Fig. 7). Chemoheterotrophy and aerobic chemoheterotrophy were more prominent in young soils (0–40-year groups) and tended to decline with increasing soil age. These predicted functions were associated with a diverse set of heterotrophic taxa, including members of Vicinamibacteraceae, *Polaromonas, Bradyrhizobium, Massilia*, and several genera within Nitrosomonadaceae.

In contrast, predicted cellulolytic functions were more frequently assigned in Gaunt Creek fluvial deposit and stable soils at Jackson Bay. These assignments were primarily linked to ASVs affiliated with the actinobacterial genus *Acidothermus*.

Predicted methane-cycling functions showed variation across the chronosequence. Methanotrophy was relatively more frequently assigned in intermediate-aged soils (40–80- year groups) and less frequently in both younger (20–40-year group) and both oldest soil groups (>100-year Gaunt Creek fluvial deposit and stable soils at Jackson Bay). These assignments were largely associated with ASVs classified as *Ca*. Methylomirabilis, which reached up to 0.5% relative abundance in the 60–80-year group. Canonical aerobic methanotrophic lineages represented only a minor fraction of the community (< 0.1% relative abundance). Predicted methanogenic functions were mainly associated with ASVs assigned to uncultured Methanomassiliicoccales, reaching ∼1% relative abundance in the 40–60-year group, while other methanogenic lineages were rare.

Nitrogen-cycling functions were detected across all soil age categories. Predicted nitrogen fixation showed relatively higher representation in the 20–60-year groups compared to the youngest and oldest soils. Predicted nitrification and aerobic ammonia oxidation were more frequently assigned in the youngest soils (0–20-year group) and tended to decrease with soil age. In contrast, predicted denitrification showed higher relative representation in older soils and reference sites.

Functions related to oxygenic phototrophy and sulfur cycling were detected at low relative abundances and occurred inconsistently across samples, without clear or consistent age-related patterns.

## Discussion

Soil chronosequences provide a valuable framework for investigating how microbial communities and geochemical properties co-develop during ecosystem formation. While chronosequence studies have traditionally focused on long-term pedogenesis spanning centuries to millennia, during which soils reach climax or retrogressive stages (Vitousek and Farrington 1997), the most pronounced biogeochemical transformations typically occur within the first century of soil development (Singh et al. 2001, Błońska et al. 2018, Trejos-Espeleta et al. 2024). These early stages are particularly important because microbial communities establish the foundation for nutrient cycling and soil development (Rasigraf and Wagner 2022).

Natural short-term chronosequences are comparatively rare but occur in geomorphically active landscapes such as the landslide-prone western Southern Alps of New Zealand (Korup 2004, Allen et al. 2011). Unlike anthropogenic restoration chronosequences, landslide deposits develop under natural disturbance regimes while remaining embedded within an intact temperate rainforest, providing an opportunity to investigate microbial succession during the first decades of soil formation.

### Microbial community structure

Grouping landslide deposits into 20-year age class intervals revealed a gradual decline in bacterial richness and Shannon diversity with increasing soil age, although significant differences became apparent only between the youngest (0–20-year group) and the oldest chronosequence stages (80–100-year group). Similar declines in bacterial diversity have been reported for the Franz Josef glacier chronosequence (Jangid et al. 2013), another primary succession system from New Zealand’s western Southern Alps with comparable climatic conditions and related parent material (Adams et al. 2009). Comparable trends have also been described in temperate forest chronosequences (Wang et al. 2022, Amen et al. 2025), although opposite patterns have been reported from subtropical secondary forests (Ren et al. 2019), indicating that successional trajectories remain ecosystem-specific.

Community composition changed more gradually than alpha diversity. Although NMDS ordination showed substantial overlap among age groups, pairwise PERMANOVA analyses restricted to the landslide chronosequence detected significant compositional differences between all age classes. Effect sizes were moderate, indicating progressive rather than abrupt community turnover during the first century of soil development. While sampling location also explained part of the observed variation, age-related differences remained significant throughout the chronosequence, supporting microbial succession as a consistent ecological pattern across the studied rainforest landscape.

The observed successional trajectory appears to be driven primarily by progressive environmental filtering. Distance-based redundancy analysis (dbRDA) indicated that the measured environmental variables collectively explained 18.98% of the variation in microbial community composition, with age category, TOC, SO_4_²⁻, and Ca²⁺ emerging as significant predictors. Importantly, variation partitioning revealed that both soil developmental stage and soil chemistry contributed to community structuring, with partially independent fractions attributable to age (4.2%) and environmental variables (5.2%), as well as a small shared component (0.6%), indicating that much of the environmental signal is intrinsically linked to soil development itself.

Young landslide deposits were characterized by low organic carbon availability and highly heterogeneous physicochemical conditions, whereas older soils accumulated organic matter and developed more stable geochemical conditions, including consistently acidic pH. The strong contribution of TOC within the constrained multivariate model supports the interpretation that organic matter accumulation is a key axis of microbial habitat differentiation during pedogenesis. However, the partitioning results also suggest that age-related processes extend beyond measured chemistry alone, likely integrating additional unmeasured factors such as microclimate buffering, substrate complexity, and plant–soil feedbacks. Similar shifts in environmental filtering have been proposed for other chronosequences, where increasing organic matter accumulation and soil development promote deterministic community assembly (Wardle et al. 2004, Tripathi et al. 2018).

Consistent with this interpretation, niche breadth analysis revealed a strong predominance of specialist taxa and no true generalists across the chronosequence. Rather than being structured by broadly adapted taxa, microbial communities became increasingly dominated by organisms occupying relatively narrow ecological niches. This pattern supports the view that progressive soil development strengthens environmental selection and promotes niche differentiation, as reported from other soil chronosequences (Bahram et al. 2018, Chen et al. 2021, Rodriguez et al. 2022).

#### Bacterial community composition

Bacterial communities across all sampled soils were dominated by a consistent set of cosmopolitan soil phyla, including Acidobacteriota, Actinobacteriota, Chloroflexi, Methylomirabilota, Planctomycetota, Pseudomonadota, and Verrucomicrobiota, which together accounted for the majority of the prokaryotic community (Fig. 4A; Fierer et al. 2007, Bergmann et al. 2011, Kaboré et al. 2020). The presence of these lineages across all age classes, including the youngest deposits, indicates the establishment of a broad core microbiome early in soil development, likely inherited from mobilized soil and regolith material during landslide emplacement.

Pseudomonadota were most abundant in early successional soils, particularly within the 0–40-year interval, and declined progressively in older soils and reference systems. This pattern was primarily driven by Burkholderiales and other fast-growing lineages, consistent with their role as metabolically flexible, copiotrophic taxa capable of exploiting transient and easily available carbon pools (Fierer et al. 2007, Lajoie and Kembel 2019, Li et al. 2021). Similar patterns have been reported in early successional and post-disturbance soils (Nemergut et al. 2007, Rime et al. 2016). In contrast, Alphaproteobacteria, particularly Rhizobiales, were most abundant in older, stable soils and positively correlated with total organic carbon (TOC) (Supplementary Fig. 6). This aligns with studies showing that Alphaproteobacteria dominate mature forest soils and are associated with decomposition of complex organic matter (Dukunde et al. 2019, Yang et al. 2022).

Several other dominant phyla displayed clear successional trends. Acidobacteriota, Chloroflexi, Planctomycetota, and Verrucomicrobiota increased in relative abundance with soil age, reaching highest values in older landslide deposits and stable soils (Zhou et al. 2003, Fierer et al. 2007, Bergmann et al. 2011, Davis et al. 2011, Dunfield et al. 2024). While these taxa are often associated with oligotrophic lifestyles, their distribution in this system appears more closely linked to the development of complex and chemically heterogeneous organic matter pools rather than simple carbon limitation. This interpretation is supported by the observed positive relationship between TOC and microbial abundance and by previous studies showing similar successional patterns in temperate and alpine chronosequences (Nemergut et al. 2007, Feng et al. 2023, Trejos-Espeleta et al. 2024).

Functional predictions based on FAPROTAX further support this successional framework (Louca et al. 2016). Early-stage soils were characterized by higher relative representation of chemoheterotrophic and aerobic heterotrophic functions, consistent with rapid exploitation of labile carbon inputs immediately following landslide disturbance. These functions declined with soil age, while functions associated with degradation of more complex organic substrates increased in older soils, indicating a functional shift toward processing of more recalcitrant carbon compounds during ecosystem development.

Taken together, bacterial community composition in landslide soils reflects a gradual transition from disturbance-influenced, metabolically flexible pioneer assemblages toward increasingly specialized communities structured by soil development processes. This successional shift is tightly linked to progressive accumulation and transformation of soil organic matter, as well as the stabilizing physicochemical environment that develops during the first century of pedogenesis.

#### Archaeal community composition

Although bacteria dominated overall community composition, archaea were consistently present across all landslide soils, highlighting their ubiquity even in early successional mineral soils. Archaeal relative abundances remained low compared to bacteria but showed a general increase with soil age, while exhibiting a consistent negative relationship with soil TOC content. This pattern suggests that archaeal distribution is more strongly associated with soil developmental stage and organic matter quality than with total carbon availability.

Across all sampled soils, the archaeal community was dominated by members of Thermoproteota, particularly Nitrosotaleales, Nitrosopumilales, and Gagatemarchaeaceae. Nitrosotaleales and Nitrosopumilales were relatively more abundant in early successional soils, consistent with their well-established role as oligotrophic ammonia-oxidizing archaea widely distributed in acidic soils (Leininger et al. 2006, Nicol et al. 2008, Prosser and Nicol 2012). In contrast, Gagatemarchaeaceae increased progressively with soil age, reaching their highest abundances in older landslide deposits and reference soils, consistent with previous chronosequence studies in similar temperate systems (Nicol et al. 2006, Turner et al. 2017).

The ecological interpretation of this successional shift has recently changed. Genomic analyses demonstrate that Gagatemarchaeaceae, unlike canonical ammonia-oxidizing members of Thermoproteota, lack the genetic potential for ammonia oxidation and instead encode pathways consistent with aerobic heterotrophic metabolism and the utilization of diverse organic compounds (Sheridan et al. 2023 ). Their increasing abundance with soil age therefore likely reflects adaptation to progressively more complex organic matter pools rather than enhanced nitrification, supporting a role in soil organic matter turnover during pedogenesis.

The second major archaeal component consisted of Thermoplasmatota, including Methanomassiliicoccales and *Ca*. Poseidoniales. Methanomassiliicoccales were present across most soils but were reduced in stable reference sites, consistent with their known occurrence in diverse soil environments as methyl-dependent methanogens (Söllinger and Urich 2019, Jurado et al. 2020). *Ca*. Poseidoniales archaea, while typically associated with marine systems, have also been reported in saline and terrestrial soils, including deserts and coastal soils (Finstad et al. 2017, Wan et al. 2021), suggesting that their occurrence in landslide soils may reflect hydrological connectivity and transient microsite conditions. Their consistently low abundance suggests a minor but persistent role within the soil archaeal community.

Nanoarchaeota, represented mainly by Woesearchaeales, showed a similar pattern to *Ca*. Poseidoniales, with slightly higher representation in younger soils and uniformly low abundance across all samples, consistent with their widespread but low-abundance distribution in soils globally (Zhang et al. 2015, Rinke et al. 2019).

Overall, archaeal community structure reflects a transition from disturbance-influenced assemblages in early soils toward communities increasingly dominated by Gagatemarchaeaceae and other oligotrophic lineages in later successional stages. The absence of a positive relationship between archaeal abundance and TOC, together with the consistent negative correlation across archaeal taxa, supports the interpretation that archaeal community assembly is primarily controlled by soil developmental stage and substrate quality rather than bulk carbon availability. In particular, the higher relative abundance of Gagatemarchaeaceae in older soils is consistent with their recently revised metabolic potential for aerobic degradation of organic compounds, suggesting an important role in soil carbon transformation during late-stage pedogenesis (Sheridan et al. 2023).

#### Vertical distribution of microbial communities in landslide soils

Depth profiles across all sampled landslide deposits did not show a consistent shift in microbial community composition with increasing depth (Supplementary Fig. 7). In a few cases, such as the 0–20-year deposit at Hare Mare Creek, deeper soils (> 300 cm) formed a distinct cluster, but this pattern was not observed in other deposits. A more robust separation emerged between fragmented bedrock material and organic soil patches that developed on the deposit surface. These surface patches consistently clustered apart from the underlying material and were characterized by higher TOC concentrations, one of the strongest predictors of microbial community structure.

The fine-scaled depth profile at the 12.5-year-old landslide at Gaunt Creek showed no significant stratification in either microbial composition or TOC concentrations (Fig. 6). Surprisingly, TOC remained low even in the upper 0–20 cm layer, despite the rapid topsoil development typically observed in New Zealand (Sparling et al. 2003, Schomakers et al. 2017). Slow carbon accumulation may be driven by the steep slope (> 30°), which can reduce plant establishment by limiting seed fixation and persistence (Cerdà and García-Fayos 1997, Bochet et al. 2009). Although landslide deposits tend to hold more moisture than surrounding hillslopes (Wicki et al. 2023), microsite instability, rapid runoff, and sparse vegetation likely constrain the incorporation of organic matter. Microbial assemblies are strongly influenced by soil nutrient content (Knelman et al. 2014, Tian et al. 2017, Guo et al. 2024), and C and N accumulation in soils is strongly dependent on the establishment of vegetation (Jobbágy and Jackson 2000). Hence, delayed plant colonization in landslide soils may slow microbial community shifts.

As an exception to inconsistent vertical changes in carbon and microbial communities in all of our sampled depth profiles, one fine-scaled profile collected at the central toe of the Hare Mare Creek landslide revealed significant differences in microbial-community composition and between the upper and lower 40 cm that correlate with changes in TOC and 16S rRNA gene copy numbers (Fig. 6). Interestingly, TOC and 16S rRNA abundance increased below 40 cm, which deviates from typical surface-driven soil development patterns. The initial landslide event occurred 23.5 ± 8.5 years before sampling, but it was classified into the 0–20-year group due to a major reactivation event in 2013/2014, which effectively reset the surface age to 4.5 years. Whereas the exact depth affected by reactivation cannot be determined without high-resolution elevation data, patterns of TOC, 16S rRNA copy numbers, and microbial composition suggest that the material below 40 cm may incorporate material from the original landslide surface that underwent pedogenesis for 10–25 years before being buried and re-worked by a re-activation. We note that this horizon with elevated TOC could not be detected in other depth profiles from the periphery of this landslide deposit, suggesting that the reworking was inhomogeneous across the deposit.

Reactivation events are common at younger sites with sparse vegetation and unstable slopes (Samia et al. 2017). Their variable magnitude can cause uneven reworking, deposition, and burial of older surfaces, introducing complexity to TOC patterns. Whether vertical patterns such as in the Hare Mare Creek deposit are widespread across reactivated landslides, and how long these signals persist in buried material, remains to be determined.

## Conclusions

This study demonstrates that microbial community assembly in landslide-affected soils of the western Southern Alps is strongly structured by early pedogenic development and underlying lithology. Despite high initial heterogeneity, a consistent successional signal emerged within the first century of soil formation, indicating progressive but gradual microbial turnover rather than abrupt community replacement.

Across the chronosequence, microbial diversity tended to decline with soil age, while community composition shifted in association with increasing organic carbon content and progressive soil acidification. These environmental gradients were also reflected in multivariate analyses, with total organic carbon and selected geochemical variables emerging as key drivers of community structure.

Taxonomically, early-stage soils were characterized by fast-growing and metabolically flexible bacterial assemblages, whereas older soils were increasingly dominated by more specialized taxa associated with stable and carbon-enriched conditions. Archaeal communities showed a complementary pattern, with a shift toward heterotrophic Gagatearchaeota in later successional stages and generally low abundance of methanogenic groups across all soils.

Depth-resolved analyses highlighted that landslide soils may preserve the legacy of reactivation events, as initial surfaces become buried under newly deposited material. Such events are likely common on young, unstable slopes and create heterogeneity in organic carbon and microbial distributions. Profiling soil depth for biogeochemical and microbial signals may therefore provide a valuable tool for reconstructing the history of landslide deposits.

Overall, our results highlight that microbial succession in landslide soils is tightly coupled to rapid changes in soil chemistry and organic matter accumulation during the first decades of ecosystem development. These findings emphasize the importance of early pedogenic processes in shaping microbial community assembly and biogeochemical potential in dynamic, disturbance-prone landscapes.

## Supplementary Material

fiag086_Supplemental_Files
