# Supplementary material for: From disturbance to resilience: early microbial community establishment in landslide soils of New Zealand
Source: FEMS Microbiol Ecol. 2026 Aug 3;102(8):fiag086. doi: 10.1093/femsec/fiag086 (PMC13459131; doi:10.1093/femsec/fiag086)
Supplement: Supplementary file 1 [file fiag086_supplemental_files.zip › List of Supplementary Figures.docx]

**List of Supplementary Figures**

**Supplementary Figure 1** | Soil pH and total organic carbon (TOC) across the landslide chronosequence. (A) Relationship between soil pH and total organic carbon (TOC, %) across all sampled soils, with points coloured by age category. (B) Distribution of soil pH across landslide age categories (0–20-, 20–40-, 40–60-, 60–80-, 80–100-, >100-year Gaunt Creek fluvial deposit). Boxes represent interquartile ranges, whiskers extend to 1.5× IQR, and individual observations are shown as jittered points.

**Supplementary Figure 2** | Microbial community composition across soil age groups.
(A) Non-metric multidimensional scaling (NMDS) ordination based on Bray–Curtis dissimilarities of Hellinger-transformed community data. Points represent individual samples coloured by soil age group; ellipses indicate 95% confidence intervals around group centroids. (B) Pairwise PERMANOVA results based on Bray–Curtis dissimilarities, shown as a heatmap of between-group differences across soil age categories along the chronosequence gradient. P-values were adjusted for multiple comparisons using Bonferroni correction, and significant differences are indicated by black asterisks. Blue asterisks indicate significant differences in multivariate dispersion (β-dispersion, tested using *betadisper* and permutation tests). Groups comprise 0–20-, 20–40-, 40–60-, 60–80-, and 80–100-year landslide soils, the >100-year Gaunt Creek fluvial deposit, and stable soils from Jackson Creek (“st. soil”).

**Supplementary Figure 3** | Correlations between environmental parameters and microbial community composition. Pearson correlation heatmap showing relationships between measured environmental parameters and SRS-normalized relative abundances of the most abundant bacterial and archaeal phyla across sampled soils. Correlations were calculated using Pearson’s correlation coefficient. P-values were adjusted for multiple testing using the Benjamini–Hochberg false discovery rate (FDR) correction. Only FDR-adjusted significant correlations are indicated by asterisks: ****p* ≤ 0.001, ***p* ≤ 0.05, **p* ≤ 0.1.

**Supplementary Figure 4** | Specialist and intermediate taxa across soil age groups.
Heatmap showing mean relative abundances of taxa identified as specialists or intermediates based on niche breadth (Levins’ B index). Rows represent agglomerated genera, annotated with the soil age group in which each taxon shows specialist preference (e.g. 0–20), ASV identifier, and genus name. Colour intensity indicates mean relative abundance across soil age groups, highlighting age-dependent distributions of niche-associated taxa. Groups comprise 0–20-, 20–40-, 40–60-, 60–80-, and 80–100-year landslide soils, the >100-year Gaunt Creek fluvial deposit, and stable soils from Jackson Creek (“st. soil”).

**Supplementary Figure 5** | Principal coordinate analysis of microbial communities in vertical soil profiles. Principal coordinate analysis (PCoA) of microbial communities in fine-scale vertical soil profiles from a 4.5-year-old landslide (Hare Mare Creek) and a 12.5-year-old landslide (Gaunt Creek). Points are coloured by soil depth and shaped by site. PCoA was based on Jaccard distance metrics.

**Supplementary Figure 6** | Correlations between environmental parameters and microbial community composition. Pearson correlation heatmap showing relationships between measured environmental parameters and SRS-normalized relative abundances of the most abundant bacterial and archaeal orders across sampled soils. Correlations were calculated using Pearson’s correlation coefficient. P-values were adjusted for multiple testing using the Benjamini–Hochberg false discovery rate (FDR) correction.Only FDR-adjusted significant correlations are indicated by asterisks: ****p* ≤ 0.001, ***p* ≤ 0.05, **p* ≤ 0.1.

**Supplementary Figure 7** | Principal coordinate analysis of microbial communities across sites and substrate types. Principal coordinate analysis (PCoA) of microbial communities from individual sites, including landslide deposits, fluvial deposits, and stable soils. Points are coloured by soil depth and shaped by sampled material (fragmented bedrock and organic soil patches). PCoA was based on Jaccard distance metrics.
