# Supplementary material for: From disturbance to resilience: early microbial community establishment in landslide soils of New Zealand
Source: FEMS Microbiol Ecol. 2026 Aug 3;102(8):fiag086. doi: 10.1093/femsec/fiag086 (PMC13459131; doi:10.1093/femsec/fiag086)
Supplement: Supplementary file 1 [file fiag086_supplemental_files.zip › Supplementary_Figure_1.pdf]

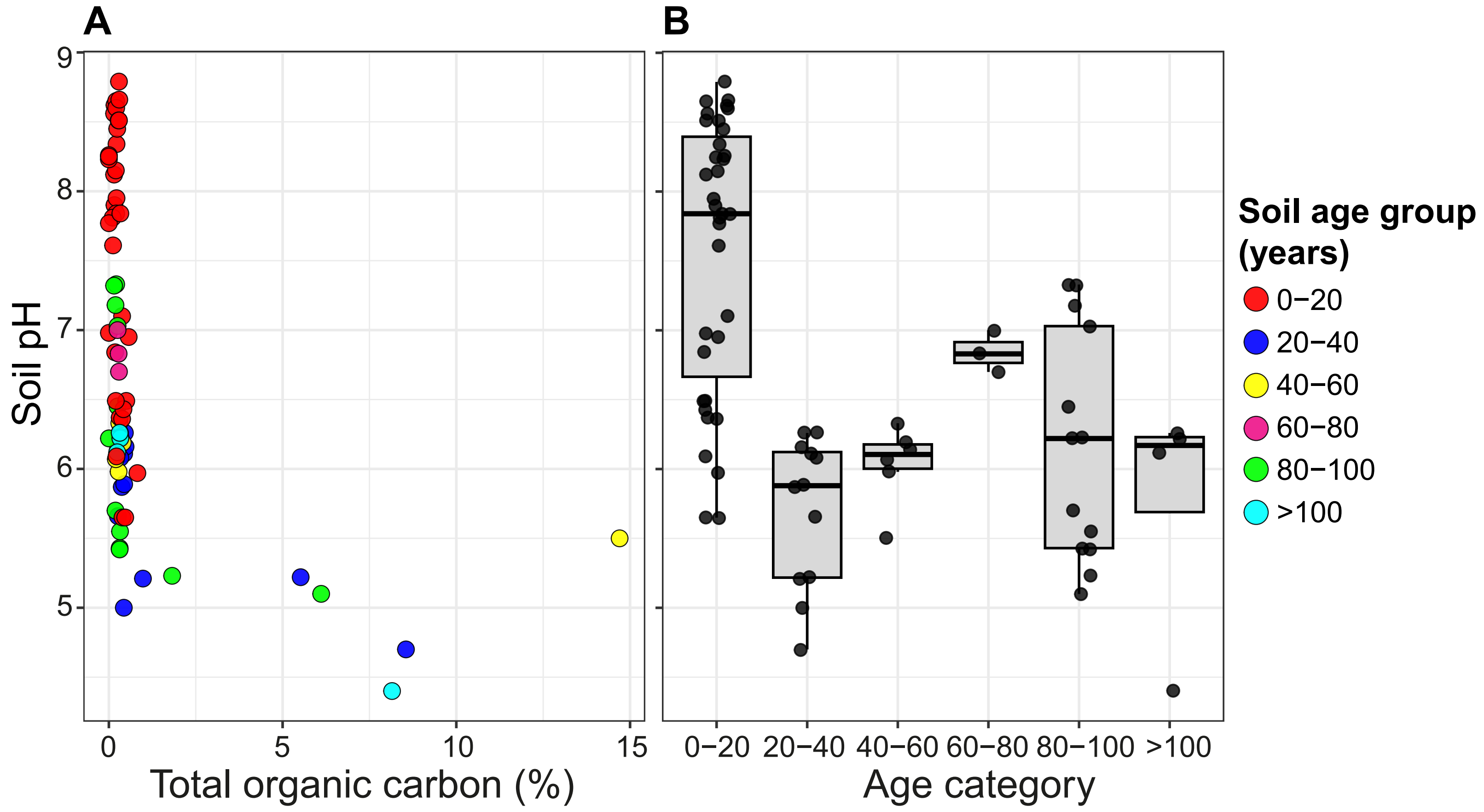
A
B
Soil pH
9
8
7
6
5
0
5
10
15
Total organic carbon (%)
0−20
20−40
40−60
60−80
80−100
>100
Age category
Soil age group (years)
0−20
20−40
40−60
60−80
80−100
>100
