# Supplementary material for: From disturbance to resilience: early microbial community establishment in landslide soils of New Zealand
Source: FEMS Microbiol Ecol. 2026 Aug 3;102(8):fiag086. doi: 10.1093/femsec/fiag086 (PMC13459131; doi:10.1093/femsec/fiag086)
Supplement: Supplementary file 1 [file fiag086_supplemental_files.zip › Supplementary_Figure_2.pdf]

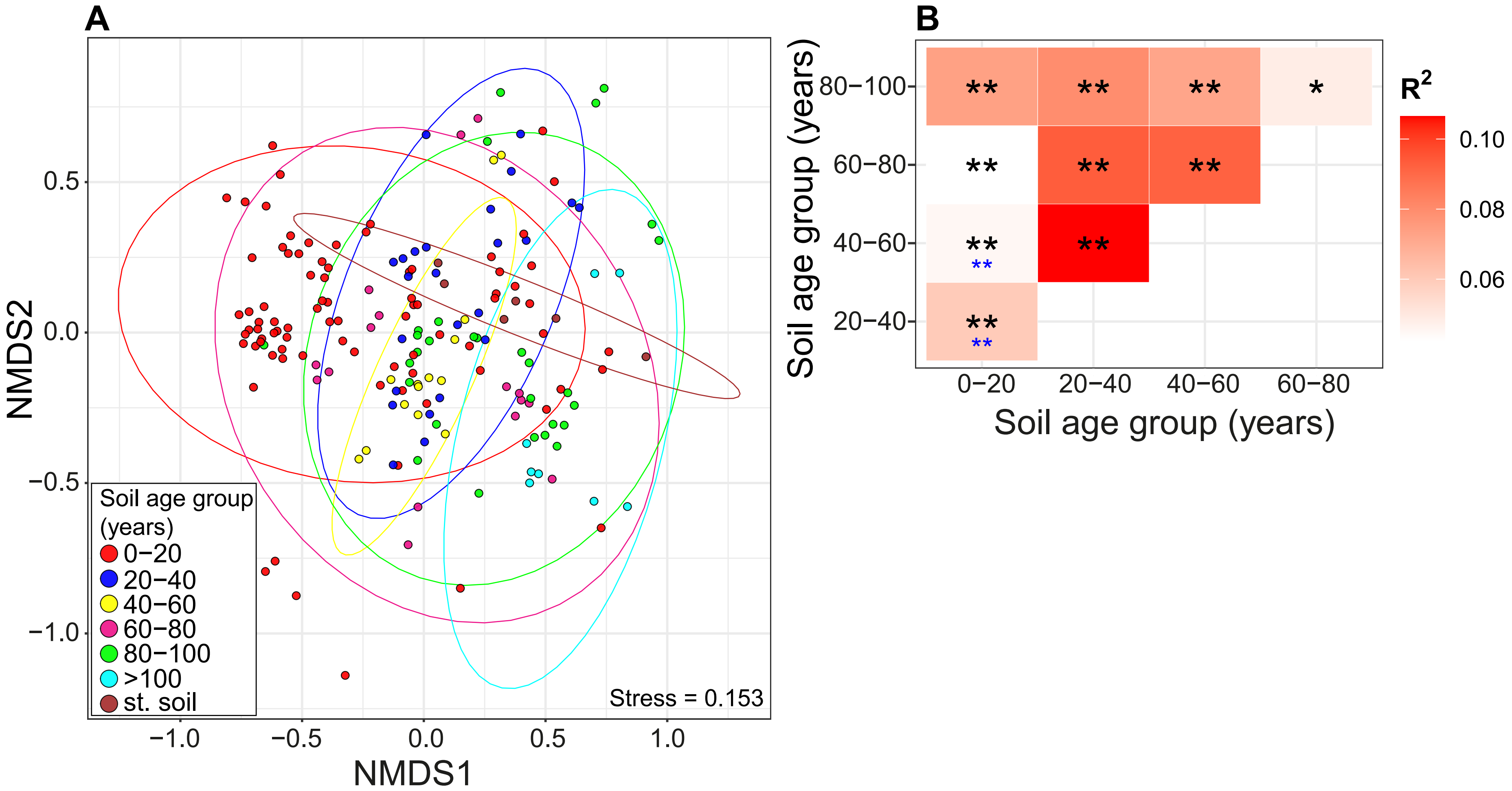
A
NMDS2
NMDS1
0.5
0.0
−0.5
−1.0
−1.0
−0.5
0.0
0.5
1.0
Soil age group (years)
0−20
20−40
40−60
60−80
80−100
>100
st. soil
Stress = 0.153
B
Soil age group (years)
80−100
60−80
40−60
20−40
0−20
20−40
40−60
60−80
R^2
0.10
0.08
0.06
**
*
