# Supplementary material for: From disturbance to resilience: early microbial community establishment in landslide soils of New Zealand
Source: FEMS Microbiol Ecol. 2026 Aug 3;102(8):fiag086. doi: 10.1093/femsec/fiag086 (PMC13459131; doi:10.1093/femsec/fiag086)
Supplement: Supplementary file 1 [file fiag086_supplemental_files.zip › Supplementary_Figure_3.pdf]

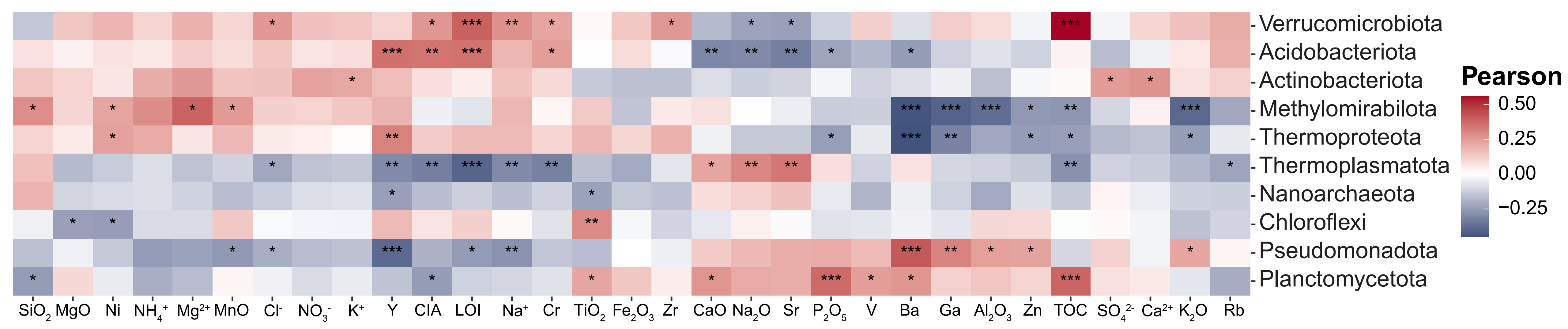
Verrucomicrobiota
Acidobacteriota
Actinobacteriota
Methylomirabilota
Thermoproteota
Thermoplasmatota
Nanoarchaeota
Chloroflexi
Pseudomonadota
Planctomycetota
SiO2
MgO
Ni
NH4+
Mg2+
MnO
Cl-
NO3-
K+
Y
CIA
LOI
Na+
Cr
TiO2
Fe2O3
Zr
CaO
Na2O
Sr
P2O5
V
Ba
Ga
Al2O3
Zn
TOC
SO42-
Ca2+
K2O
Rb
Pearson
0.50
0.25
0.00
−0.25
