# Supplementary material for: From disturbance to resilience: early microbial community establishment in landslide soils of New Zealand
Source: FEMS Microbiol Ecol. 2026 Aug 3;102(8):fiag086. doi: 10.1093/femsec/fiag086 (PMC13459131; doi:10.1093/femsec/fiag086)
Supplement: Supplementary file 1 [file fiag086_supplemental_files.zip › Supplementary_Figure_4.pdf]

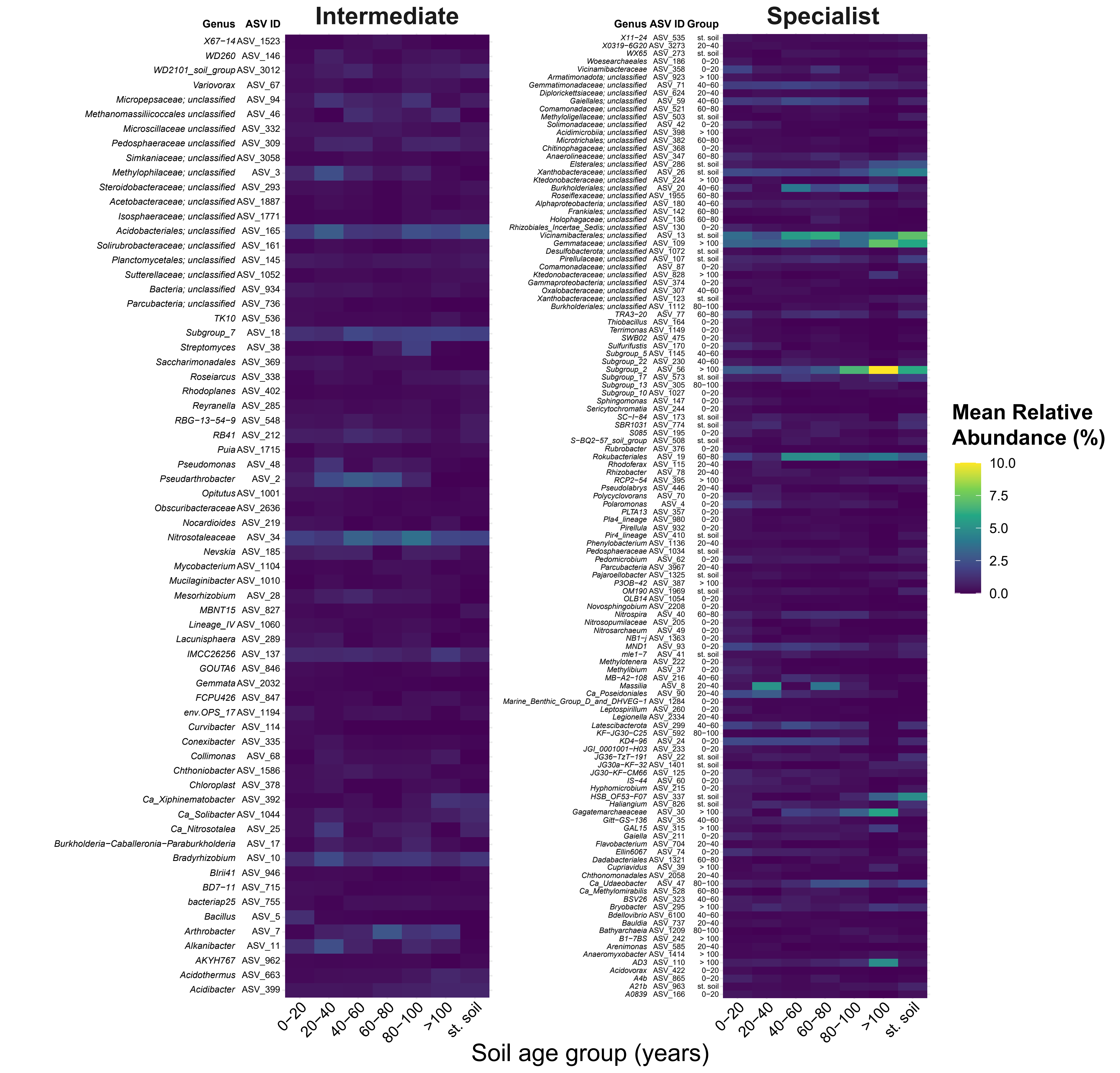
Intermediate
Genus ASV ID
X67−14 ASV_1523
WD260 ASV_146
WD2101_soil_group ASV_3012
Variovorax ASV_67
Micropepsaceae; unclassified ASV_94
Methanomassiliicoccales unclassified ASV_46
Microscillaceae unclassified ASV_332
Pedosphaeraceae unclassified ASV_309
Simkaniaceae; unclassified ASV_3058
Methylophilaceae; unclassified ASV_3
Steroidobacteraceae; unclassified ASV_293
Acetobacteraceae; unclassified ASV_1887
Isosphaeraceae; unclassified ASV_1771
Acidobacteriales; unclassified ASV_165
Solirubrobacteraceae; unclassified ASV_161
Planctomycetales; unclassified ASV_145
Sutterellaceae; unclassified ASV_1052
Bacteria; unclassified ASV_934
Parcubacteria; unclassified ASV_736
TK10 ASV_536
Subgroup_7 ASV_18
Streptomyces ASV_38
Saccharimonadales ASV_369
Roseiarcus ASV_338
Rhodoplanes ASV_402
Reyranella ASV_285
RBG−13−54−9 ASV_548
RB41 ASV_212
Puia ASV_1715
Pseudomonas ASV_48
Pseudarthrobacter ASV_2
Opitutus ASV_1001
Obscuribacteraceae ASV_2636
Nocardioides ASV_219
Nitrosotaleaceae ASV_34
Nevskia ASV_185
Mycobacterium ASV_1104
Mucilaginibacter ASV_1010
Mesorhizobium ASV_28
MBNT15 ASV_827
Lineage_IV ASV_1060
Lacunisphaera ASV_289
IMCC26256 ASV_137
GOUTA6 ASV_846
Gemmata ASV_2032
FCPU426 ASV_847
env.OPS_17 ASV_1194
Curvibacter ASV_114
Conexibacter ASV_335
Collimonas ASV_68
Chthoniobacter ASV_1586
Chloroplast ASV_378
Ca_Xiphinematobacter ASV_392
Ca_Solibacter ASV_1044
Ca_Nitrosotalea ASV_25
Burkholderia−Caballeronia−Paraburkholderia ASV_17
Bradyrhizobium ASV_10
Blrii41 ASV_946
BD7−11 ASV_715
bacteriap25 ASV_755
Bacillus ASV_5
Arthrobacter ASV_7
Alkanibacter ASV_11
AKYH767 ASV_962
Acidothermus ASV_663
Acidibacter ASV_399
0−20
20−40
40−60
60−80
80−100
>100
st. soil
Specialist
Genus ASV ID Group
X11−24 ASV_535 st. soil
X0319−6G20 ASV_3273 20−40
WX65 ASV_273 st. soil
Woesearchaeales ASV_186 0−20
Vicinamibacteraceae ASV_358 0−20
Armatimonadota; unclassified ASV_923 > 100
Gemmatimonadaceae; unclassified ASV_71 40−60
Diplorickettsiaceae; unclassified ASV_624 20−40
Gaiellales; unclassified ASV_59 40−60
Comamonadaceae; unclassified ASV_521 60−80
Methyloligellaceae; unclassified ASV_503 st. soil
Solimonadaceae; unclassified ASV_42 0−20
Acidimicrobiia; unclassified ASV_398 > 100
Microtrichales; unclassified ASV_382 60−80
Chitinophagaceae; unclassified ASV_368 0−20
Anaerolineaceae; unclassified ASV_347 60−80
Elsterales; unclassified ASV_286 st. soil
Xanthobacteraceae; unclassified ASV_26 st. soil
Ktedonobacteraceae; unclassified ASV_224 > 100
Burkholderiales; unclassified ASV_20 40−60
Roseiflexaceae; unclassified ASV_1955 60−80
Alphaproteobacteria; unclassified ASV_180 40−60
Frankiales; unclassified ASV_142 60−80
Holophagaceae; unclassified ASV_136 60−80
Rhizobiales_Incertae_Sedis; unclassified ASV_130 0−20
Vicinamibacterales; unclassified ASV_13 st. soil
Gemmataceae; unclassified ASV_109 > 100
Desulfobacterota; unclassified ASV_1072 st. soil
Pirellulaceae; unclassified ASV_107 st. soil
Comamonadaceae; unclassified ASV_87 0−20
Ktedonobacteraceae; unclassified ASV_828 > 100
Gammaproteobacteria; unclassified ASV_374 0−20
Oxalobacteraceae; unclassified ASV_307 40−60
Xanthobacteraceae; unclassified ASV_123 st. soil
Burkholderiales; unclassified ASV_1112 80−100
TRA3−20 ASV_77 60−80
Thiobacillus ASV_164 0−20
Terrimonas ASV_1149 0−20
SWB02 ASV_475 0−20
Sulfurifustis ASV_170 0−20
Subgroup_5 ASV_1145 40−60
Subgroup_22 ASV_230 40−60
Subgroup_2 ASV_56 > 100
Subgroup_17 ASV_573 st. soil
Subgroup_13 ASV_305 80−100
Subgroup_10 ASV_1027 0−20
Sphingomonas ASV_147 0−20
Sericytochromatia ASV_244 0−20
SC−I−84 ASV_173 st. soil
SBR1031 ASV_774 st. soil
S085 ASV_195 0−20
S−BQ2−57_soil_group ASV_508 st. soil
Rubrobacter ASV_376 0−20
Rokubacteriales ASV_19 60−80
Rhodoferax ASV_115 20−40
Rhizobacter ASV_78 20−40
RCP2−54 ASV_395 > 100
Pseudolabrys ASV_446 20−40
Polycyclovorans ASV_70 0−20
Polaromonas ASV_4 0−20
PLTA13 ASV_357 0−20
Pla4_lineage ASV_980 0−20
Pirellula ASV_932 0−20
Pir4_lineage ASV_410 st. soil
Phenylobacterium ASV_1136 20−40
Pedosphaeraceae ASV_1034 st. soil
Pedomicrobium ASV_62 0−20
Parcubacteria ASV_3967 20−40
Pajaroellobacter ASV_1325 st. soil
P3OB−42 ASV_387 > 100
OM190 ASV_1969 st. soil
OLB14 ASV_1054 0−20
Novosphingobium ASV_2208 0−20
Nitrospira ASV_40 60−80
Nitrosopumilaceae ASV_205 0−20
Nitrosarchaeum ASV_49 0−20
NB1−j ASV_1363 0−20
MND1 ASV_93 0−20
mle1−7 ASV_41 st. soil
Methylotenera ASV_222 0−20
Methylibium ASV_37 0−20
MB−A2−108 ASV_216 40−60
Massilia ASV_8 20−40
Ca_Poseidoniales ASV_90 20−40
Marine_Benthic_Group_D_and_DHVEG−1 ASV_1284 0−20
Leptospirillum ASV_260 0−20
Legionella ASV_2334 20−40
Latescibacterota ASV_299 40−60
KF−JG30−C25 ASV_592 80−100
KD4−96 ASV_24 0−20
JGI_0001001−H03 ASV_233 0−20
JG36−TzT−191 ASV_22 st. soil
JG30a−KF−32 ASV_1401 st. soil
JG30−KF−CM66 ASV_125 0−20
IS−44 ASV_60 0−20
Hyphomicrobium ASV_215 0−20
HSB_OF53−F07 ASV_337 st. soil
Haliangium ASV_826 st. soil
Gagatemarchaeaceae ASV_30 > 100
Gitt−GS−136 ASV_35 40−60
GAL15 ASV_315 > 100
Gaiella ASV_211 0−20
Flavobacterium ASV_704 20−40
Ellin6067 ASV_74 0−20
Dadabacteriales ASV_1321 60−80
Cupriavidus ASV_39 > 100
Chthonomonadales ASV_2058 20−40
Ca_Udaeobacter ASV_47 80−100
Ca_Methylomirabilis ASV_528 60−80
BSV26 ASV_323 40−60
Bryobacter ASV_295 > 100
Bdellovibrio ASV_6100 40−60
Bauldia ASV_737 20−40
Bathyarchaeia ASV_1209 80−100
B1−7BS ASV_242 > 100
Arenimonas ASV_585 20−40
Anaeromyxobacter ASV_1414 > 100
AD3 ASV_110 > 100
Acidovorax ASV_422 0−20
A4b ASV_865 0−20
A21b ASV_963 st. soil
A0839 ASV_166 0−20
0−20
20−40
40−60
60−80
80−100
>100
st. soil
Mean Relative Abundance (%)
10.0
7.5
5.0
2.5
0.0
Soil age group (years)
