# Supplementary material for: From disturbance to resilience: early microbial community establishment in landslide soils of New Zealand
Source: FEMS Microbiol Ecol. 2026 Aug 3;102(8):fiag086. doi: 10.1093/femsec/fiag086 (PMC13459131; doi:10.1093/femsec/fiag086)
Supplement: Supplementary file 1 [file fiag086_supplemental_files.zip › Supplementary_Figure_5.pdf]

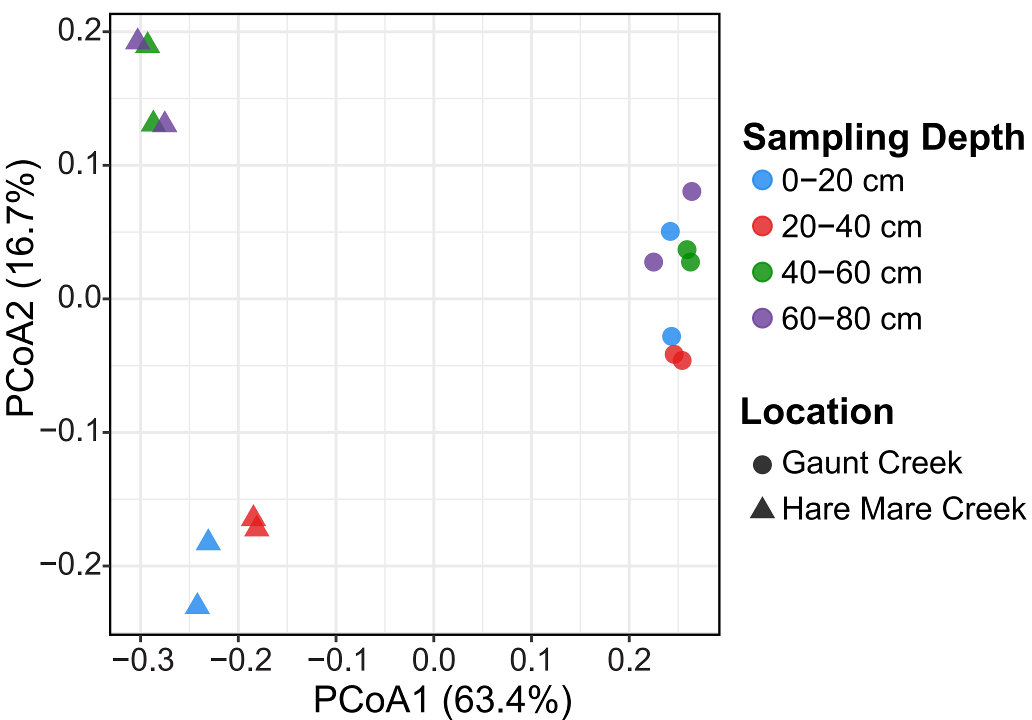
PCoA2 (16.7%)
PCoA1 (63.4%)
0.2
0.1
0.0
−0.1
−0.2
−0.3
−0.2
−0.1
0.0
0.1
0.2
Sampling Depth
0−20 cm
20−40 cm
40−60 cm
60−80 cm
Location
Gaunt Creek
Hare Mare Creek
