# Supplementary material for: From disturbance to resilience: early microbial community establishment in landslide soils of New Zealand
Source: FEMS Microbiol Ecol. 2026 Aug 3;102(8):fiag086. doi: 10.1093/femsec/fiag086 (PMC13459131; doi:10.1093/femsec/fiag086)
Supplement: Supplementary file 1 [file fiag086_supplemental_files.zip › Supplementary_Figure_6.pdf]

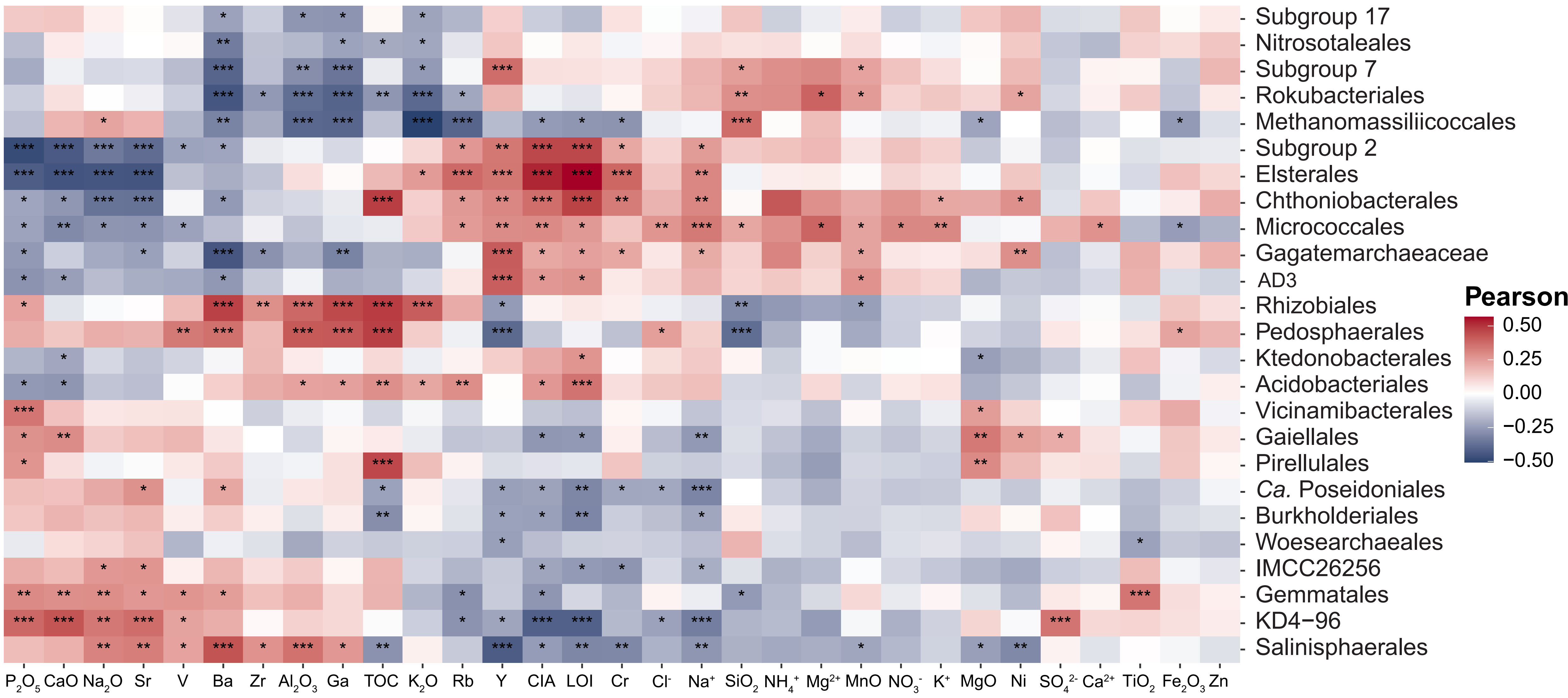

Subgroup 17
Nitrosotaleales
Subgroup 7
Rokubacteriales
Methanomassiliicoccales
Subgroup 2
Elsterales
Chthoniobacterales
Micrococcales
Gagatemarchaeaceae
AD3
Rhizobiales
Pedosphaerales
Ktedonobacterales
Acidobacteriales
Vicinamibacterales
Gaiellales
Pirellulales
Ca. Poseidoniales
Burkholderiales
Woesearchaeales
IMCC26256
Gemmatales
KD4−96
Salinisphaerales
P2O5 CaO Na2O Sr V Ba Zr Al2O3 Ga TOC K2O Rb Y CIA LOI Cr Cl- Na+ SiO2 NH4+ Mg2+ MnO NO3- K+ MgO Ni SO42- Ca2+ TiO2 Fe2O3 Zn
Pearson
0.50
0.25
0.00
−0.25
−0.50
