# Supplementary material for: From disturbance to resilience: early microbial community establishment in landslide soils of New Zealand
Source: FEMS Microbiol Ecol. 2026 Aug 3;102(8):fiag086. doi: 10.1093/femsec/fiag086 (PMC13459131; doi:10.1093/femsec/fiag086)
Supplement: Supplementary file 1 [file fiag086_supplemental_files.zip › Supplementary_Figure_7.pdf]

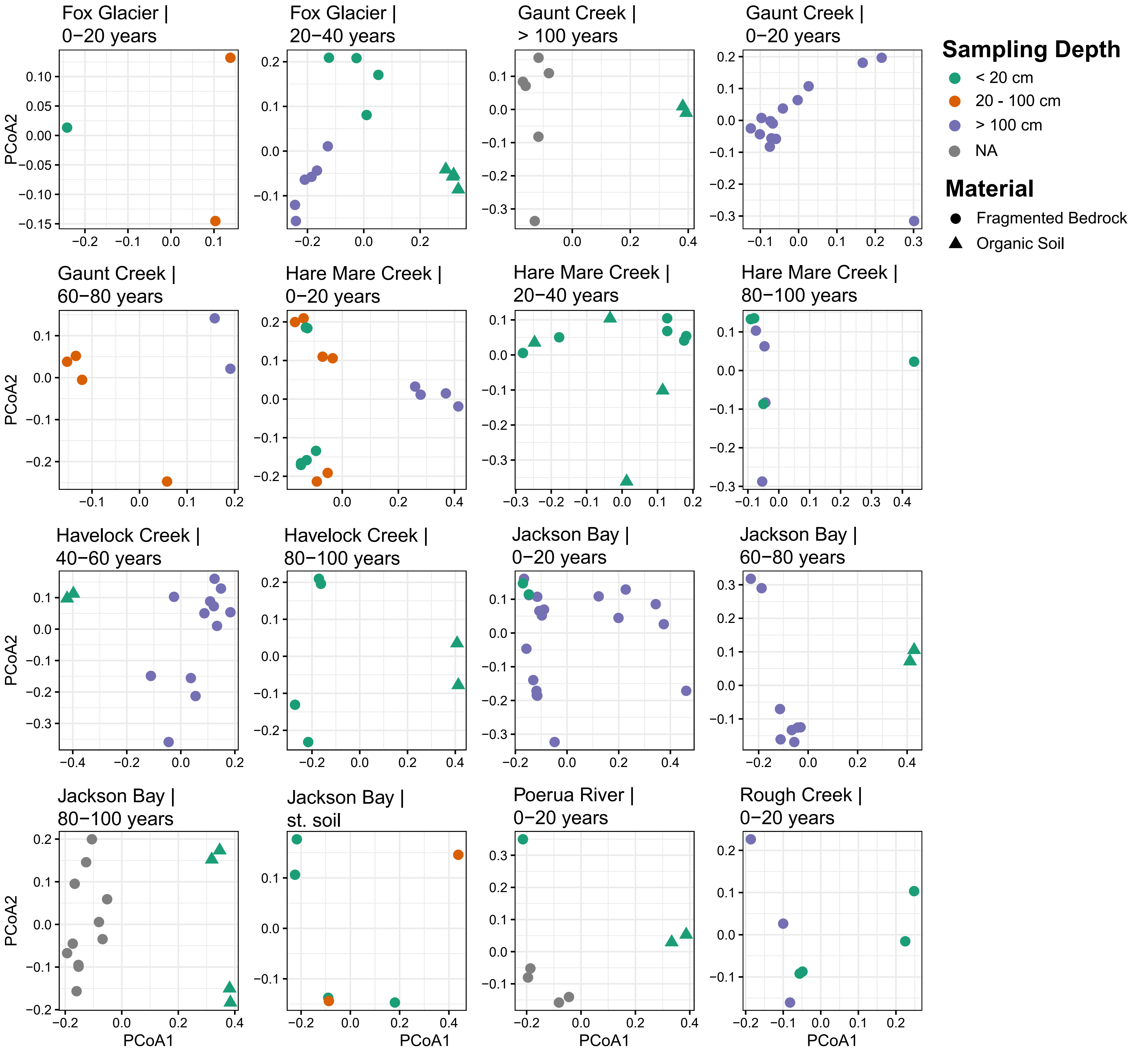

Fox Glacier | 0−20 years
Fox Glacier | 20−40 years
Gaunt Creek | > 100 years
Gaunt Creek | 0−20 years
Gaunt Creek | 60−80 years
Hare Mare Creek | 0−20 years
Hare Mare Creek | 20−40 years
Hare Mare Creek | 80−100 years
Havelock Creek | 40−60 years
Havelock Creek | 80−100 years
Jackson Bay | 0−20 years
Jackson Bay | 60−80 years
Jackson Bay | 80−100 years
Jackson Bay | st. soil
Poerua River | 0−20 years
Rough Creek | 0−20 years
PCoA1
PCoA2
Sampling Depth
< 20 cm
20 - 100 cm
> 100 cm
NA
Material
Fragmented Bedrock
Organic Soil
